# Characterization and Expression Patterns of Auxin Response Factors in Wheat

**DOI:** 10.3389/fpls.2018.01395

**Published:** 2018-09-19

**Authors:** Linyi Qiao, Wenping Zhang, Xiaoyan Li, Lei Zhang, Xiaojun Zhang, Xin Li, Huijuan Guo, Yuan Ren, Jun Zheng, Zhijian Chang

**Affiliations:** ^1^Shanxi Key Laboratory of Crop Genetics and Molecular Improvement, Key Laboratory of Crop Gene Resources and Germplasm Enhancement on Loess Plateau of the Ministry of Agriculture, Institute of Crop Science, Shanxi Academy of Agricultural Sciences, Taiyuan, China; ^2^Center for Genomics and Biotechnology, Haixia Institute of Science and Technology, Fujian Agriculture and Forestry University, Fuzhou, China; ^3^Beijing Institute of Heart Lung and Blood Vessel Diseases, Beijing Anzhen Hospital Affiliated with the Capital Medical University, Beijing, China; ^4^Department of Plant Protection, College of Agriculture, Shanxi Agricultural University, Taigu, China

**Keywords:** genomewide, ARFs, alternative splicing, expression pattern, PAML, transgenic functional verification

## Abstract

Auxin response factors (ARFs) are important transcription factors involved in both the auxin signaling pathway and the regulatory development of various plant organs. In this study, 23 TaARF members encoded by a total of 68 homeoalleles were isolated from 18 wheat chromosomes (excluding chromosome 4). The TaARFs, including their conserved domains, exon/intron structures, related microRNAs, and alternative splicing (AS) variants, were then characterized. Phylogenetic analysis revealed that members of the TaARF family share close homology with ARFs in other grass species. qRT-PCR analyses revealed that 20 TaARF members were expressed in different organs and tissues and that the expression of some members significantly differed in the roots, stems, and leaves of wheat seedlings in response to exogenous auxin treatment. Moreover, protein network analyses and co-expression results showed that TaTIR1–TaARF15/18/19–TaIAA13 may interact at both the protein and genetic levels. The results of subsequent evolutionary analyses showed that three transcripts of *TaARF15* in the A subgenome of wheat exhibited high evolutionary rate and underwent positive selection. Transgenic analyses indicated that *TaARF15-A.1* promoted the growth of roots and leaves of *Arabidopsis thaliana* and was upregulated in the overexpression plants after auxin treatment. Our results will provide reference information for subsequent research and utilization of the *TaARF* gene family.

## Introduction

Auxin signaling is key to many plant growth and developmental processes ranging from embryogenesis to senescence (Strader and Zhao, 2016; Mironova et al., 2017). Auxin response factors (ARFs), which generally consist of an amino-terminal DNA-binding domain (DBD), a middle region (MR) that functions as either an activation domain or a repression domain, and a carboxy-terminal dimerization domain (CTD), are transcription factors involved in the well-described transport inhibitor response 1/auxin signaling F-Box (TIR1/AFB) auxin signaling pathway (Boer et al., 2014; Dinesh et al., 2015). In the absence of auxin, auxin/indole acetic acid (Aux/IAA) repressor proteins bind to the CTD of ARFs and inhibit their function. When present, auxin promotes the TIR1/AFB-mediated ubiquitin–proteasome-dependent degradation of Aux/IAAs (Jing et al., 2015), thus relieving ARFs from repression and allowing them to activate or repress the expression of auxin-responsive genes; this activation or repression occurs *via* the DBD that binds to auxin response elements (AuxREs) within the promoters of target genes (Dinesh et al., 2015; Winkler et al., 2017).

In the model plant *Arabidopsis thaliana*, 23 *ARF* genes have been identified (Wei et al., 2006); several of these members, such as *AtARF1*–*8* (Ellis et al., 2005; Marin et al., 2010; Pitaksaringkarn et al., 2014; Moller et al., 2017), *AtARF10*, *AtARF16* (Yang et al., 2014), *AtARF17* (Gutierrez et al., 2009), and *AtARF19* (Galvan-Ampudia and Vernoux, 2014), are involved in regulating the morphology and growth of roots, stems, leaves, flowers, and fruits. Based on these *AtARF*s whose functions are known, homology cloning has been used to identify many *ARF*s in other species, including *OsARF8* (Yang et al., 2006), *SlARF8* (Ma et al., 2015), *GmARF8* (Wang et al., 2015, *NtARF8* (Zhu et al., 2013), and *InARF8* (Glazinska et al., 2014), all of which are homologous to *AtARF8*. In addition, whole-genome sequencing studies have led to the isolation of 25 *ARF*s from rice (Wang et al., 2007), 24 *ARF*s from sorghum (Wang et al., 2010), 31 *ARF*s from maize (Xing et al., 2011), 51 *ARF*s from soybean (Ha et al., 2013), 19 *ARF*s from tomato (Zouine et al., 2014), and 24 *ARF*s from *Medicago truncatula* (Shen et al., 2015). However, few studies have investigated this gene family in bread wheat (*Triticum aestivum* L.), one of the most widely grown crops worldwide, as this species has an enormous and complex hexaploid genome. Currently, only one wheat expressed sequence tag (GenBank No. AY902381) has been reported, which responds to aluminum stress (Liu et al., 2017).

The rapid development of sequencing and assembly technologies has led to the completion (*via* different sequencing technologies) of the draft genome of “Chinese Spring” bread wheat (Brenchley et al., 2012; Mayer et al., 2014; Zimin et al., 2017). In addition, the physical map (IWGSC, 2017^1^) as well as a high-quality genome (Clavijo et al., 2017) have been published, allowing the isolation and analysis of gene families on a genomic scale. In this study, 23 *ARF* members, with a total of 68 homeoalleles, were isolated in wheat, and the genomic location, sequence characteristics, related microRNAs, alternative splicing (AS) variants, phylogenetic relationships, and expression patterns of those *ARF*s were analyzed. In addition, the evolutionary rate and transgene function of *TaARF15-A* were verified. These results will provide reference information for subsequent research and utilization of the *TaARF* gene family.

## Materials and Methods

### Isolation and Bioinformatic Analysis of Protein Sequences

The TGACv1 collection of whole-protein sequences of “Chinese Spring” wheat was downloaded from the Ensembl database^2^. The sequences of the predicted wheat ARF proteins were obtained by retrieving the whole-protein sequence data based on the ARF family Hidden Markov Model profiles (Pfam accession number PF00931) and checking the ARF domains using the hmmsearch and hmmscan programs of the nhmmer software (Wheeler and Eddy, 2013) program, respectively; a cutoff of *E* ≤1e-5 was used. *Via* their registration number (**Supplementary Table S1**), the protein sequences of ARFs from Arabidopsis, rice and other species were downloaded directly from the NCBI database (National Center for Biotechnology Information^3^).

Multiple sequence alignments of the ARFs were performed using Clustal X (Larkin et al., 2007). Phylogenetic trees were constructed using MEGA6.0 software (Tamura et al., 2013) with the neighbor-joining method and 1000 bootstraps. The secondary structures of the protein sequences were predicted using the NPS@ server (Network Protein Sequence Analysis^4^; Combet et al., 2000). The STRING database (Search Tool for the Retrieval of Interacting Genes/Proteins^5^; Szklarczyk et al., 2015) was used to predict interactions among ARFs, Aux/IAAs, and TIR1 proteins in wheat. The TaTIR1 protein sequence was retrieved from the Ensembl database by BLASTP queries of the OsTIR1 sequence (Xia et al., 2012), and we obtained the sequences of Aux/IAA proteins from our previous research (Qiao et al., 2015).

### Characterization and Evolutionary Rate Analysis of Gene Sequences

The coding DNA sequences (CDS), AS variants, and genomic sequences of the *TaARF* gene family members were extracted (*via* their protein accession number) from the Ensembl database. In reference to previous studies (Kim et al., 2007; Kaur et al., 2017), the AS events of *TaARFs* were identified by comparing the genetic structure of AS variants with the assumed wild-type sequence (generally the AS variant numbered 1) so that each variant was identified as one of the five categories: intron retention (IR), alternative 5′ splicing (A5SS), alternative 3′ splicing (A3SS), exon skipping (ES), or mutually exclusive exon (MXE). The position information of these members was determined by using the RefSeq v1.0 iteration of the wheat whole genome, which was downloaded from the IWGSC database^6^, after which the *TaARF*s were assigned to their corresponding chromosomes. The sequences of wheat microRNAs that may regulate *TaARF*s were obtained by retrieving tae-miR target sequence data (downloaded from the Ensembl database) using *TaARF* CDS as queries with a similarity >90%. Then, a professional small RNA target analysis server, psRNATarget (Dai et al., 2018), was used for the validation of bioinformatics with maximum expectation set as zero. Gene structures were determined using GSDS 2.0 (Gene Structure Display Server^7^; Hu et al., 2015). Putative promoter regions (2000 bp upstream of the start codon) of *TaIAA*s were obtained from their genomic sequences, and the AuxREs that bind ARF proteins, TGTCTC (Ulmasov et al., 1995) and TGTCGG (Boer et al., 2014), were identified by manually scanning the *TaIAA* promoters.

The *ARF* codon sequences were used for constructing phylogenetic trees and in subsequent calculations. The ratio of non-synonymous substitutions per non-synonymous site to synonymous substitutions per synonymous site (ω value) of branches was computed by using the maximum likelihood method of the branch model in the PAML 4.4 software package (Yang, 2007). If the likelihood ratio test (LRT) indicated that the ω value of a specified branch significantly differed from the constant ω value across all branches, that branch was used for the next positive selection analysis of the branch-site model using the Bayes empirical Bayes method described by Yang et al. (2005). The *TuARF*, *AetARF*, and *TdARF* sequences used for the evolutionary analysis were obtained by using BLASTN to query the *TaARF15* CDS against the predicted gene sequences of *Triticum urartu* (Ling et al., 2013), *Aegilops tauschii* (Zhao et al., 2017), and *Triticum dicoccoides* (Avni et al., 2017), three ancestors of wheat.

### Auxin Treatment, qRT-PCR, and Digital Gene Expression

With respect to auxin treatment, the wheat cultivar “Chinese Spring” was planted under a long-day photoperiod (15 h of light, 9 h of darkness). Seedlings with three fully opened leaves were sprayed with 10 μM α-naphthylacetic acid (α-NAA) solution or distilled water (mock treatment), as described previously (Zhao et al., 2013). The roots, stems, and leaves of seedlings were sampled at 0, 0.5, 1.5, and 3 h after auxin treatment, and at each time point, samples from three plants were pooled, after which they were stored at -80°C. The total RNA was extracted using an RNA extraction kit (Tiangen Biotech, China) and reverse-transcribed into cDNA with an M-MLV reverse transcription kit (Invitrogen, United States). qRT-PCR was subsequently performed using SYBR Premix Ex Taq II (Takara Bio Inc., China), and each reaction was repeated three times; *GADPH* and *TaAux/IAA1* (Singla et al., 2006) served as the internal and external control, respectively. The qRT-PCR primers listed in **Supplementary Table S2** were designed based on the consensus sequence of homeoalleles for every *TaARF* member, and the specific primers for each AS variant of *TaARF15-A* were also designed. The qRT-PCR data were analyzed using the fold-change (Navarro et al., 2006) and the 2^-ΔΔ*C*_t_^ (Livak and Schmittgen, 2001) methods. Statistical analyses of the differences between the treatment group and the control group were performed by using *t*-tests (tails = 2, type = 1).

The expression data of *TaARFs* in different organs at different growth stages of Chinese Spring (Ramírez-González et al., 2018) were obtained from the expVIP database^8^ (Borrill et al., 2016) and then viewed as a heat map using the MeV tool (Multiple Experiment Viewer^9^).

### Plant Transformation

To generate *TaARF15-A1* overexpression plants, the primers 15A1-F and 15A1-R (**Supplementary Table S2**) were used to amplify the coding sequence of the wheat cDNA, after which the sequence under the control of the CaMV 35S promoter was inserted into a pBI121 binary vector using Gateway BP Clonase enzyme mix (Invitrogen, United States). The construct was then transformed into Arabidopsis (Col-0) by *Agrobacterium tumefaciens* strain GV3101 (Herrera-Estrella et al., 2005) *via* the floral dip method. The transformed lines were first selected on half-strength Murashige and Skoog medium that contained 50 mg L^-1^ kanamycin (Ahmed et al., 2012) and then screened by PCR. The resistant seedlings were subsequently transferred to a mixture of soil and vermiculite (1:1) at 22°C under a 16/8-h light/dark cycle with 70% relative humidity, after which homozygous lines were generated by self-fertilization. Plants from the F_3_ generation and wild-type Arabidopsis were used for morphological comparison. qRT-PCR was then used to detect the expression of *TaARF15-A.1* in the transgenic plants. The total RNA was isolated from leaves of 20-day-old *TaARF15-A1* overexpression plants and wild-type plants at 0, 0.5, and 2 h after 10 μM auxin treatment, and *ACTIN2* served as an endogenous control. qRT-PCR for each line was based on three independent biological replicates.

## Results

### Distribution and Domain of the TaARF Family

**FIGURE 1:**
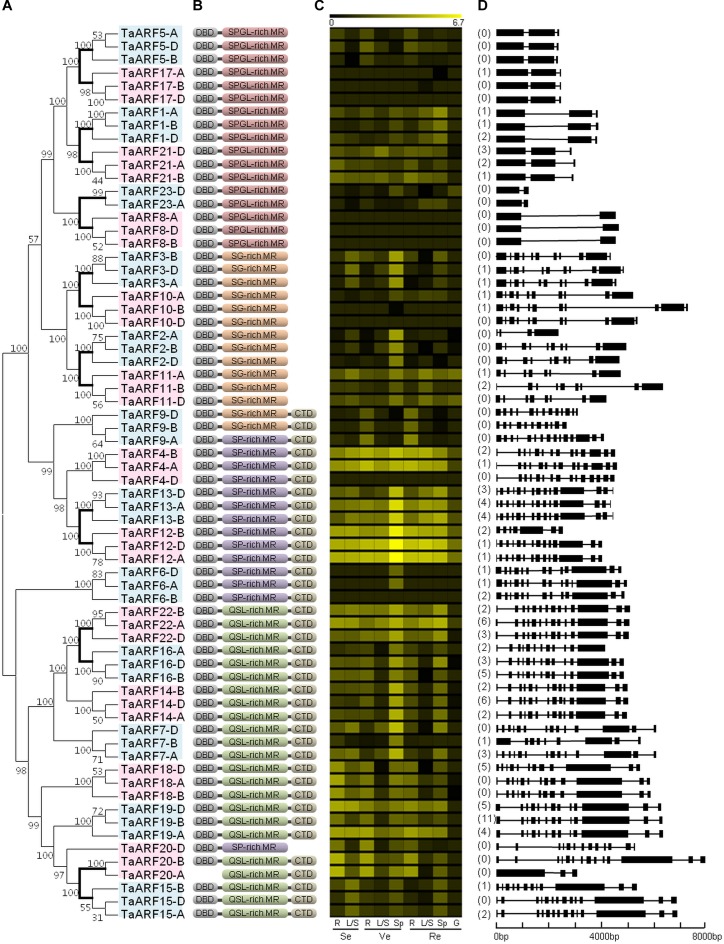
Phylogenetic relationships, domains, *in silico* expression profiling, and gene structure of ARFs in wheat. **(A)** Phylogenetic tree of TaARFs constructed from the complete alignment of 68 wheat ARF protein sequences by the neighbor-joining method with 1000 bootstrap replicates using MEGA 6.0. The bootstrap scores are indicated on the nodes, and the 23 TaARF members, all of which are coded by homeoalleles, are indicated in blue or pink boxes. The branch of paralogous TaARF members is shown in bold. **(B)** Conserved domains of TaARFs. DBD, DNA-binding domain; CTD, C-terminal dimerization domain; MR, middle region; S, serine; P, proline; G, glycine; L, leucine; Q, glutamine. **(C)** *In silico* expression profiling of TaARF homeoalleles in different organs at different growth stages of Chinese Spring wheat. The expression data were generated from the expVIP database (http://www.wheat-expression.com/). The color scale at the top represents the expression values: black indicates low levels of transcript abundance, and yellow indicates high levels. R, root; L/S, leaf/stem; Sp, spike; G, grain; Se, seedling stage; Ve, vegetative stage; Re, reproductive stage. **(D)** Exon/intron structures in the CDS of *TaARF* genes. The number of AS variants is listed before each gene. For genes that represent predicted AS variants, the variant with the highest *in silico* expression level has been selected for the gene structure analysis. Exons are represented by black boxes and introns by black lines. The size of exons and introns can be estimated using the scale at the bottom.

By retrieving wheat protein sequence data and examining the domains, we detected 68 full-length ARF proteins, which were subsequently used to construct an unrooted tree for revealing phylogenetic relationships. The results showed that these wheat ARF proteins could be divided into 23 groups (**Figure 1A**); each group containing two or three homeoalleles from the wheat A, B, and D subgenomes was regarded as a member of the ARF family in wheat (TaARF family). These 23 TaARF members were distributed across all chromosomes except 4A, 4B, and 4D and were named TaARF1–TaARF23 based on their chromosome position (**Supplementary Figure S1**). The largest number of members (TaARF18–23) were distributed across homologous chromosome 7, while only one member (TaARF14) was found on homologous chromosome 5. With the exception of TaARF23, every TaARF member had three homeoalleles.

The length of the 68 TaARF proteins varied from 354 amino acids (AAs) for TaARF23-D to 1174 AA for TaARF20-B (**Supplementary Table S3**). With the exception of TaARF20-A, every TaARF protein contained a DBD for binding the promoter region of target genes. Thirty-eight TaARF proteins contained a CTD, suggesting that those proteins may be inhibited by Aux/IAA proteins. In addition, all of the 68 TaARF proteins contained a MR: 17 of the proteins contained an SPGL-rich MR, 14 contained an SG-rich MR, 14 contained an SP-rich MR, and 23 contained a QSL-rich MR (**Figure 1B** and **Supplementary Figure S2**). The first three types may suppress the expression of target genes, while the last one may promote it (Guilfoyle and Hagen, 2007). Furthermore, 34 TaARF proteins contained all three domains.

### MicroRNAs, AS Events, and Expression Profiling Related to *TaARF* Genes

The full lengths of the *TaARF* genes varied from 1119 bp for *TaARF23-A* to 7915 bp for *TaARF20-B*, and the number of introns ranged from zero to 14 (**Supplementary Table S3**). The homeoalleles for most *TaARF* members, including *TaARF1*, *3*, *5*, *6*, *8*, *10*, *13*, *14*, *17*, *19*, *21*, *22*, and *23*, showed analogous exon/intron structures (**Figure 1D**). Furthermore, high sequence similarities (93.76–100%) were observed between several target sequences of wheat microRNAs and the sequences of *TaARF1*, *5*, *7*, *17*, *21*, and *22*. Finally, *TaARF1-A*, *-B*, and *-D* and *TaARF17-B* were predicted to be the targets for tae-miR160 (**Supplementary Figure S1** and **Supplementary Table S4**).

**FIGURE 2:**
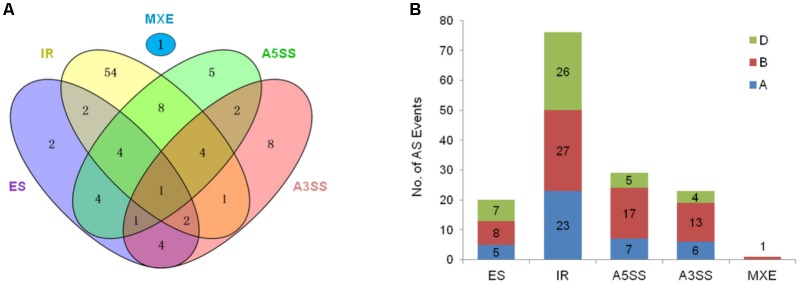
Summary of AS in TaARFs. **(A)** Five types of AS in 103 splice variants from *TaARF* homeoalleles. The number of splice variants is represented in each intersection of the Venn diagram. IR, intron retention; ES, exon skipping; A5SS, alternative 5′ splicing site; A3SS, alternative 3′ splicing site; MXE, mutually exclusive exon. **(B)** One hundred forty-nine AS events occurring in 103 splice variants. The AS events that occurred in the A, B, and D subgenomes are indicated in blue, red, and green, respectively.

Furthermore, 41 homeoalleles of 17 *TaARF* members were found to have 103 AS variants; 16 homeoalleles had only one AS variant, while *TaARF19-B* had the most (12) AS variants (**Figure 1D** and **Supplementary Table S3**). Furthermore, 33 of the 103 AS variants resulted from two or more AS events, resulting in a total of 149 AS events. Among these AS events, 76 were IR events, which were the most abundant (51.7%), followed by A5SS (29), A3SS (23), ES (20), and MXE (1) events (**Figure 2A**). On the whole, the AS variants in the wheat B subgenome presented the most AS events among all AS types (**Figure 2B**).

In addition, the *in silico* expression data revealed that *TaARF4*, *12*, *13*, and *22* were highly expressed during seedling, vegetative, and reproductive stages in wheat (**Figure 1C**). The expression levels of *TaARF5* and *TaARF9* were highest in the root, while *TaARF2* and *TaARF3* were expressed highly in the spike.

### Phylogeny of ARFs

We constructed a phylogenetic tree of the protein sequences of 68 TaARFs, 23 AtARFs, 25 OsARFs, and 14 ARFs with known functions from other species. These ARFs can be classified into nine groups: Groups I–IX (**Supplementary Figure S3**). TaARFs were distributed among all groups, and each TaARF member has an orthologous OsARF.

Based on homology cloning in previous studies, many ARFs were shown to have functions similar to those of their orthologous AtARFs, such as SlARF2 (Ren et al., 2017)–AtARF2 (Ellis et al., 2005), MtARF3 (Peng et al., 2017)–AtARF3 (Marin et al., 2010), SlARF4 (Sagar et al., 2013)–AtARF4 (Marin et al., 2010), GmARF8 (Wang et al., 2015)–AtARF8 (Pitaksaringkarn et al., 2014), SlARF9 (de Jong et al., 2015)–AtARF9, and BnARF18 (Liu et al., 2015)–AtARF18, and each of these ortholog pairs was located in the same branch (**Supplementary Figure S3**). Thus, TaARFs may also have functions similar to those of their orthologous OsARFs in the same branch. For example, TaARF9–OsARF1 (Shen et al., 2013) from Group V, TaARF15–OsARF5 (Indoliya et al., 2016 and TaARF20–OsARF19 (Zhang et al., 2015) from GroupVII, TaARF7–OsARF12 (Yang et al., 2006) from Group VIII, and TaARF16–OsARF6 (Meng et al., 2009) from Group IX, may have similar functions.

### Response of *TaARF*s to Exogenous Auxin

**FIGURE 3:**
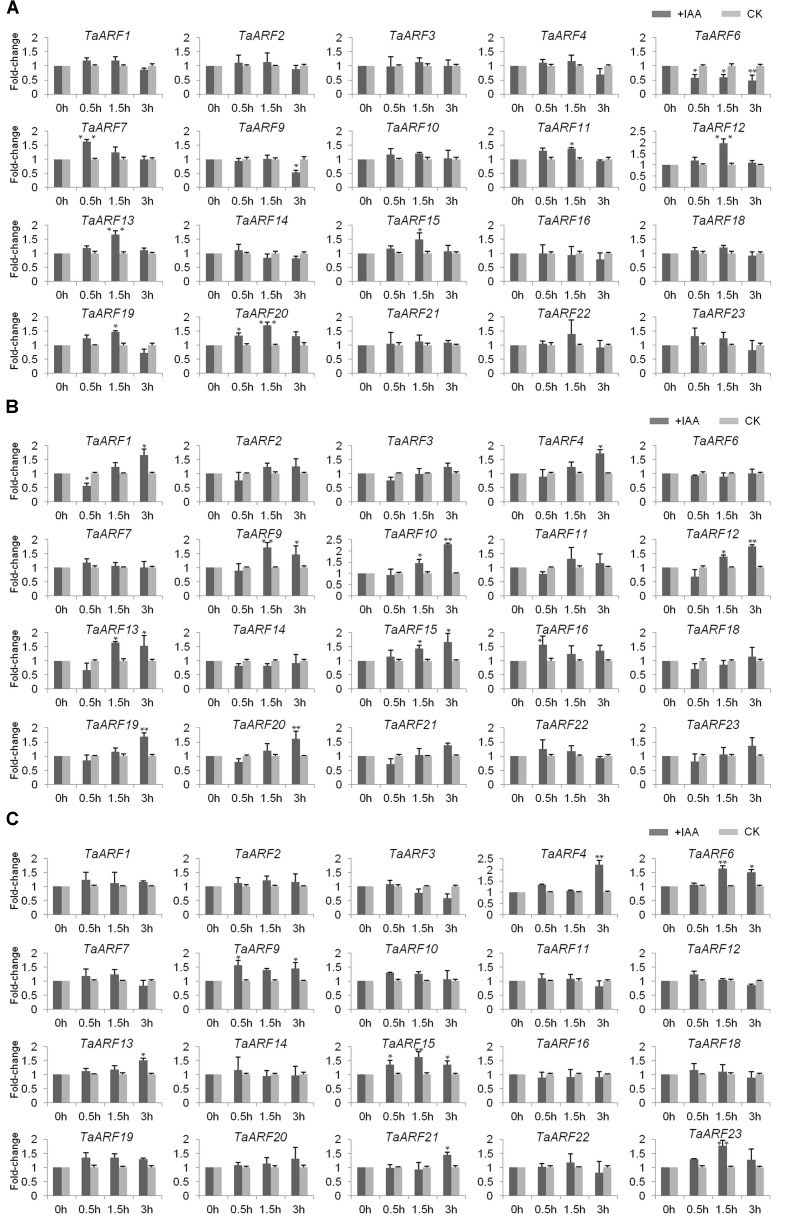
Induction (fold change) of TaARF genes in response to exogenous auxin stimuli in the roots **(A)**, stems **(B)**, and leaves **(C)** of wheat seedlings. The roots, stems and leaves of wheat cultivar “Chinese Spring” at the three-leaf stage were harvested at 0, 0.5, 1.5, and 3 HAT; the plants were treated with 10 μM α-NAA solution or distilled water (mock treatment). The relative expression level of each gene was measured three times and then normalized to that of the GADPH gene, after which the levels were analyzed using the fold-change method. Paired *t*-tests were used to detect significant differences in relative expression levels of genes between the auxin treatment and the mock treatment at each time point. The asterisks indicate significant differences, and the error bars indicate the SD.

qRT-PCR analysis of *TaARF*s in the roots, stems, and leaves of wheat seedlings revealed no expression of *TaARF5*, *TaARF7*, or *TaARF17*. The response of the other 20 *TaARF* members to exogenous auxin stimuli was investigated in wheat seedlings treated with 10 μM NAA. qRT-PCR analysis revealed that seven *TaARF* members (*TaARF7*, *11*, *12*, *13*, *15*, *19*, and *20*) were upregulated and that two members, *TaARF6* and *9*, were downregulated in the roots in response to exogenous auxin treatment (**Figure 3A**). Among those *TaARF*s, the expression levels of *TaARF11*, *12*, *13*, *15*, *19*, and *20* increased gradually within 1.5 h after treatment (HAT) but decreased later, and the expression of *TaARF6* was downregulated across all time points. Moreover, in wheat stems, the expression levels of 10 genes (*TaARF1*, *4*, *9*, *10*, *12*, *13*, *15*, *16*, *19*, and *20*) were significantly upregulated by auxin (**Figure 3B**). Among those genes, the expression levels of *TaARF1*, *4*, *10*, *12*, *19*, and *20* were downregulated within the first 0.5 HAT but were continuously upregulated after 1.5–3 HAT, while the expression levels of *TaARF9* and *13* were lower at 3 HAT than at 1.5 HAT; the expression of *TaARF15* was upregulated across all time points. In addition, in wheat leaves, the expression levels of seven genes, *TaARF4*, *6*, *9*, *13*, *15*, *21*, and *23*, were significantly upregulated in response to auxin (**Figure 3C**), and *TaARF4-21* and *TaARF15-23* exhibited similar expression patterns in response to auxin. Overall, in the roots, stems, and leaves of wheat seedling, *TaARF9*, *13*, and *15* responded significantly to auxin treatment.

In addition, the expression level of external control *TaAux/IAA1* was similar to that reported in a previous study (Singla et al., 2006), indicating that the results are reliable (**Supplementary Figure S4**).

### Prediction of Protein Interactions Among TaARFs, TaIAAs, and TaTIR1

**FIGURE 4:**
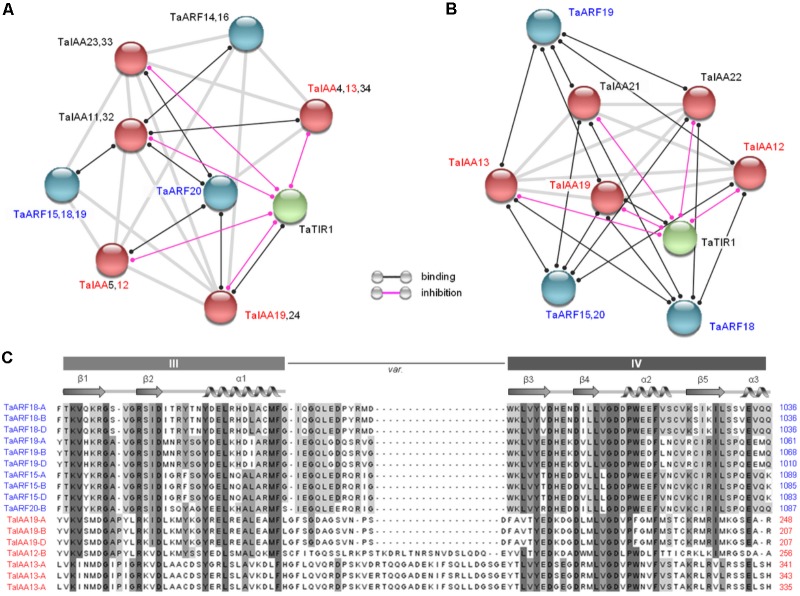
Prediction of protein interactions among TaARFs, TaIAAs, and TaTIR1. **(A,B)** Interaction networks of 20 TaARF members, 25 TaIAA members, and TaTIR1 expressed in wheat based on the interaction models of Arabidopsis and rice. Proteins for which interactions were not detected are not shown. The line colors indicate the types of interactions, which are listed in the bottom. **(C)** Residues predicted to participate in ARF and IAA protein interactions. The sequence alignment of TaARF and TaIAA proteins revealed canonical CTD domain features, including the conserved domain III and domain IV. The variable linker region is indicated by var. The α structures are shown by a spiral, and the β structures are shown by arrows.

Based on the Arabidopsis and rice interaction network models, 46 proteins expressed in wheat, including 20 TaARF members, 25 TaIAA members (Qiao et al., 2015), and one TaTIR1, were subjected to an interaction analysis. The results showed that four closely related TaARF members (TaARF15, 18, 19, and 20; **Figure 1A**) could bind to TaIAA12, 13, and 19 in both models and that TaTIR1 could also bind to TaIAA proteins and inhibit their function (**Figures 4A,B**); these results are consistent with the TIR1/AFB–IAA–ARF interaction model in the auxin signaling pathway (Boer et al., 2014; Dinesh et al., 2015). The clustering results showed that the common domain III and domain IV of the CTD of TaARF15, 18, 19, and 20 as well as those of TaIAA12, 13, and 19 showed high sequence similarity, which suggests the occurrence of a similar secondary structure that facilitates protein interactions (**Figure 4C**).

### Expression Pattern of *TaTIR1-TaARF15/19/20-TaIAA13*

**FIGURE 5:**
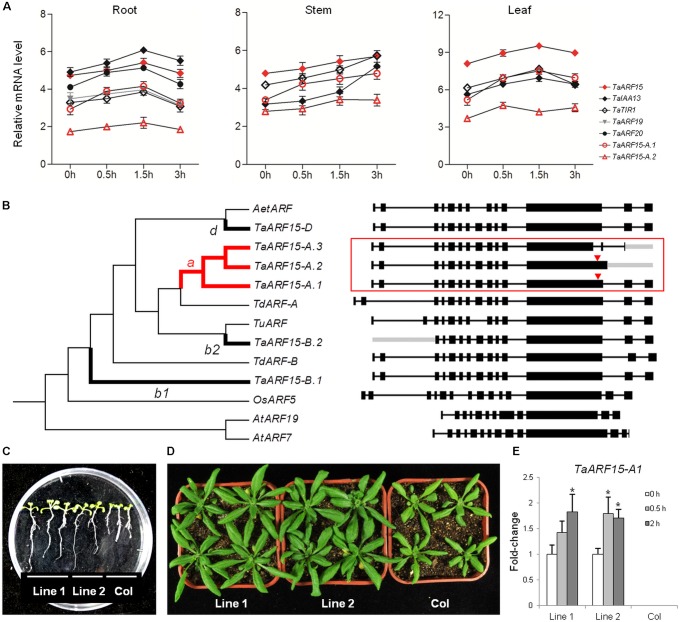
Co-expression, evolutionary, and functional analysis of TaARF15. **(A)** Auxin-related genes expressed simultaneously with TaARF15 in the roots, stems, and leaves of wheat cultivar “Chinese Spring” at the seedling stage. The qRT-PCR data were processed by the 2^-ΔΔ*C*_t_^ method, and GADPH served as the control. **(B)** Phylogenetic analyses of the six transcribed sequences of *TaARF15* as well as those of its homologs in *T. urartu* (*TuARF*, TRIUR3_19224), *Aegilops speltoides* (*AetARF*, AEGTA09095), *T. dicoccoides* (*TdARF-A*, TRIDC6AG014880.2; *TdARF-B*, TRIDC6BG020370.14), and rice (*OsARF5*, Os02g04810); *AtARF7* (AT5G20730.3) and *AtARF19* (AT1G19220.1) served as out branches. The exon/intron structures are listed on the right. The branches with *TaARF15-A*, *B*, and *D* are shown as thick lines and labeled with letters a, b1/b2, or c, respectively, and the ω values were calculated in accordance with the two-ratio model. The branch that underwent positive selection is labeled in red, and the positive selection sites of sequences are marked with red triangles. **(C,D)** Morphology of the roots and leaves of 35S::*TaARF15-A.1* lines and Col, the latter of which represents wild-type Arabidopsis plants. **(E)** Relative expression levels of *TaARF15-A.1* in both Col and homozygous F_3_ transgenic lines. The total RNA was isolated from 20-day-old leaves of Arabidopsis plants at 0, 0.5, and 2 h after 10 μM auxin solution treatment. *ACTIN2* served as an endogenous control.

At the protein level, by interacting with CTDs, Aux/IAAs inhibit ARFs. However, at the gene expression level, *Aux/IAA*s are regulated by *ARF*s because *Aux/IAA*s are the target genes of *ARF*s. Previous transcriptomic research in rice has shown that the expression levels of *TIR1* and most *ARF* genes increased in response to exogenous auxin stimulation, which subsequently regulated the expression of downstream *Aux/IAA*s (Indoliya et al., 2016). In this study, in response to auxin treatment, the expression levels of seven of eight proteins that may interact were analyzed; TaARF18 was not chosen because its response to auxin was not significant (**Figure 3**). The results showed that the expression trends of *TaTIR1*, *TaIAA13*, *TaARF15*, *TaARF19*, and *TaARF20* were consistent in wheat roots and that the expression patterns of *TaTIR1*, *TaIAA13*, and *TaARF15* were similar not only in the stems but also in the leaves (**Figure 5A**). In addition, the *TaIAA13* promoter contains AuxRE elements that are the binding sites for the DBD of ARF proteins (**Supplementary Figure S5**). Therefore, we inferred that *TaTIR1*–*TaARF15/19/20*–*TaIAA13* could also interact at the gene expression level.

### Evolutionary Rate Analysis of the Transcripts of *TaARF15*

On the whole, in response to auxin treatment, *TaTIR1*–*TaARF15*–*TaIAA13* showed the same expression trends in the roots, stems and leaves. In addition to those of *TaARF15-A*, *TaARF15-B*, and *TaARF15-D*, the transcripts of *TaARF15* can exist as two or one additional AS variant in the A and B subgenomes, respectively. These six transcripts of *TaARF15* and their orthologs in both wheat ancestral species and rice were used to construct a phylogenetic tree. The result showed that *ARF* genes from wheat and its ancestors clustered into the same group (**Figure 5B**). Among those genes, *TaARF15-D* is located in the same branch as its ortholog *AetARF* in *A. tauschii*, which is the donor of the wheat D subgenome. Furthermore, *TaARF15-A* is located in the same branch as its orthologs *TdARF-A* and *TuARF* from the ancestral species of the A subgenome. However, *TaARF15-B* is distantly related to its ancestral ortholog *TdARF-B*.

Based on the two-ratio model, the evolutionary rates of *TaARF15-A.1*–*3* (ωa = 0.66) and *TaARF15-B.1* (ωb1 = 0.99) were significantly higher than those of the background branch (ω0 = 0.17–0.18, **Supplementary Table S5**). Additional analyses revealed that only branch-an underwent positive selection; the specific site was the 717L–736S segment of the *TaARF15-A.1* protein-coding sequence, which is located on the exon 12 of the gene. *TaARF15-A.2* also contains a positive selection site, but its translation is terminated prematurely because of an IR event within intron 12. In addition, *TaARF15-A.3* lost a positive selection site in exon 12 because of an A5SS event. These examples may represent techniques used by wheat to regulate genes in different organs or stages.

### Overexpression of *TaARF15-A1* in Arabidopsis

qRT-PCR analysis for three AS variants of *TaARF15* showed that *TaARF15-A.1* was more highly expressed than *TaARF15-A.2*, while the expression of *TaARF15-A.3* was not detected. Because of the response to exogenous auxin and the more complete gene structure among *TaARF15-A* transcripts, *TaARF15-A.1* was transformed into Arabidopsis for functional verification. The results showed that the root length of the overexpression lines were significantly higher than those of the Col control lines (**Figure 5C**). Additionally, the leaf area of the overexpression lines exceeded those of the control lines (**Figure 5D**). Furthermore, qRT-PCR showed that *TaARF15-A.1* was upregulated in the overexpression plants in response to exogenous auxin treatment. Thus, it can be inferred that *TaARF15-A.1* participates in the development of roots and leaves during the vegetative growth stage of the plants (**Figure 5E**).

## Discussion

### Distribution and Evolution of Members of the ARF Family in Wheat and Other Plant Species

In this study, 23 members of the ARF family were isolated from the hexaploid wheat genome, which is closely related to that of its ancestral species, which include *T. urartu* (21), *A. tauschii* (23), and *Triticum turgidum* (25); gramineous grasses such as barley (25), *Brachypodium distachyon* (28), and rice (25, Wang et al., 2007); and the dicotyledon *A. thaliana* (23, Wei et al., 2006), and each ARF member exhibited good homology among grass species (**Supplementary Figure S6**). Phylogenetic analyses (**Supplementary Figures S3**, **S6**) revealed the poor sequence homology between TaARFs and AtARFs, which indicated that the ARF family differentiated after the divergence of mono- and dicotyledons (∼130 MYA, D’Hont et al., 2012) and that further specific differentiation occurred in Arabidopsis and gramineous plants. Interestingly, there are no ARF members on chromosome 4 in wheat, *T. urartu*, *A. tauschii*, *T. turgidum*, or barley. Therefore, the ARF family evolutionary progress at the stage during which the Triceae diverged from their grass ancestors (10–95 MYA, Pont et al., 2013) warrants further study.

There are eight pairs of paralogs in the TaARF family (**Figure 1A**). Among those paralogs, TaARF12-13 are both located on the long arm of wheat chromosome 3 at a close genomic distance, suggesting that those genes may have undergone a tandem duplication event. TaARF12-13 had homologous gene pairs in allied Triceae species but only one common ortholog in *A. thaliana*, rice, and *B. distachyon*, indicating that the tandem duplication occurred after the divergence of the Triceae ancestor (∼10 MYA, Pont et al., 2013). The remaining seven paralog pairs may have undergone segmental duplication events. Among these paralogs, TaARF1-21 has a homologous gene-pair, AtARF10-16, in *A. thaliana*, suggesting that this duplication event occurred the earliest, before the monocot–dicot divergence (∼130 MYA, D’Hont et al., 2012); however, the origins of TaARF2-11, TaARF3-10, TaARF5-17, TaARF8-23, TaARF15-20, and TaARF16-22 may occurred after the divergence. In addition, some redundant genes produced during genome doubling and duplication events may be recombined or modified, causing loss of function; these modifications include changes in structural variation, domains, and gene expression regulation as well as gradual loss (Chen, 2007; Otto, 2007). In this study, *TaARF2-A* and *TaARF9-B* lacked some exons, while TaARF20-A and TaARF20-D lacked a DBD and a CTD, respectively. Thus, these genes may suffer from the same lost fate as did *TaARF23-B*.

### AS in the TaARF Family

Alternative splicing is important for increasing the diversity and adaptability of plants (Matlin et al., 2005). In the hexaploid wheat genome, approximately 31% of the predicted coding genes have AS variants, among which IR events were the prevalent AS event (34%), followed by A3SS (27%), ES (20%), A5SS (19%), and MXE (0.04%) events (Clavijo et al., 2017). In this study, 103 AS variants were identified from 41 sequences of 17 *TaARF* members, and a total of 149 AS events, including IR (51.0%), A3SS (15.4%), ES (13.4%), A5SS (19.5%), and MXE (0.7%) events, occurred. IRs still represented the most common AS event in the *TaARF* splicing variants, and 18 of 77 IR events resulted in premature termination codons (PTCs). These PTC transcripts are often recognized and degraded by the nonsense-mediated mRNA decay (NMD) mechanism to avoid cell toxicity resulting from the accumulation of truncated protein products (Lykke-Andersen and Jensen, 2015); occasionally, these PTC transcripts encode shortened protein products that have new structures and functions (Romero et al., 2006). In addition, 17 of the 149 AS events occurred in the 5′-untranslated (UTR) region; if located in the regulatory protein binding site, the AS sites in this region can lead to altered expression of the *TaARF* gene. Similarly, 10 AS events occurred in the 3′-UTR region; these occurrences may also affect the microRNA- or long non-coding RNA (lncRNA)-based regulation of *TaARF* expression (Hughes, 2006).

### Expression Patterns of *TaARF* Members

Since the homeoalleles of most tri-genes exhibited similar expression levels (Pfeifer et al., 2014), we used universal genomic primers to analyze the expression of each *TaARF* member. The results showed that three *TaARF* members, *TaARF5*, *7*, and *17*, were not expressed, but the remaining members were all expressed in the roots, stems and leaves of wheat seedlings. Among the *TaARF* members, *TaARF9* and *13* in Group IV and *TaARF15* in Group VI both significantly responded to exogenous auxin. Since other *ARF*s in the same group, including *AtARF2*, *SlARF2*, *OsARF1*, and *OsARF24* in Group IV as well as *AtARF7*, *AtARF19*, *OsARF19*, *SlARF19*, and *OsARF5* in Group VI, were confirmed to be involved in the regulatory development of plant roots, stems, and leaves (Ellis et al., 2005; Inukai et al., 2005; Sakamoto and Inukai, 2013; Galvan-Ampudia and Vernoux, 2014; Ma et al., 2015; Zhang et al., 2015; Indoliya et al., 2016; Ren et al., 2017), it is speculated that *TaARF9*, *13*, and *15* also have similar functions. In addition, the *in silico* expression data revealed that in Group IV, *TaARF9* showed a high expression level in roots and *TaARF12* as well as *TaARF13* were highly expressed during seedlings, vegetative and reproductive stages in wheat. Moreover, *OsARF1*, the homologous gene of *TaARF9*, promoted the response of primary roots, lateral roots, and root hair to auxin (Shen et al., 2013; numbered as *OsARF16*); *AtARF2* (Ellis et al., 2005; Okushima et al., 2005; Lim et al., 2010), *SlARF2* (Xu et al., 2016; Ren et al., 2017), and *ZmARF25* (von Behrens et al., 2011; Li et al., 2014), in the same subgroup as *TaARF12* and *TaARF13*, were all confirmed to be involved in the regulation various growth stages. Therefore, *TaARF9*, *12*, and *13* may also have similar functions. Furthermore, *TaARF13* has a very close relationship with the expressed sequence tag AY902381 (**Supplementary Figure S7**), the only *ARF* sequence reported in wheat (Liu et al., 2017).

In addition to the temporal and spatial specificity of *TaARF* members and their induction by exogenous hormones, the expression of *TaARF* members may also be regulated by microRNA, which is a complex processes. For example, *TaARF17* is predicted to be the target gene of tae-miR160; thus, the lack of expression of *TaARF17* in the roots, stems, and leaves of wheat seedlings in this study is most likely due to the inhibition of miRNA. Next, we will focus on the impact of AS and microRNAs on the expression levels of some *TaARF* members.

### *TaARF15-A.1* May Be Involved in the Regulation of Roots and Leaves

*TaARF15* is an ortholog of *OsARF5*, which regulates the development of rice at different stages (Indoliya et al., 2016). In this study, *TaARF15* was expressed in the roots, stems, and leaves of wheat seedling, and its expression levels significantly differed in response to exogenous auxin treatment. Because stems are the major organ involved in polar transport and are relatively insensitive to auxin, *ARF*s could be constantly upregulated by auxin stimuli. Therefore, the expression level of *TaARF15* continuously increased in wheat stems. In the roots and leaves, however, the expression level of *TaARF15* continuously increased during the first 1.5 HAT but decreased at 3 HAT, suggesting that a negative autoregulatory feedback loop (de Jong et al., 2009) occurs in the roots and leaves sensitive to auxin treatment, causing the expression of *TaARF15* to gradually return to its initial level. In the above process, the expression patterns of the genes *TaARF15* and *TaTIR1* and the downstream gene *TaIAA13* were consistent. Because the TaARF15 protein has a QSL-MR, which may promote downstream gene expression, as well as a DBD that can bind with the AuxRE of the *TaIAA13* promoter, we speculated that *TaIAA13* is the target gene of TaARF19. The network prediction indicated that there is also a protein-level interaction involving TaTIR1–TaIAA13–TaARF15. Furthermore, *TaARF15* can express a total of six transcripts, in which *TaARF15-A.1*–*3* from the A subgenome exhibited a high evolutionary rate, and positive selection sites were detected in *TaARF15.* In addition, *TaARF15-A.1*, which has a more complete gene structure and can better respond to exogenous auxin than *TaARF15-A.2* and -*A.3*, was transferred to *A. thaliana*. Compared with the wild-type plants, the transgenic plants had longer roots and greater leaf area, and *TaARF15-A.1* could respond to exogenous auxin, which meant that *TaARF15-A.1* may participate in the regulatory development of the roots and leaves. The function of the remaining five transcripts needs further study.

## Conclusion

The TaARF family has a total of 23 members, and each member except two *TaARF* members may be the targets of tae-miR160. Seventeen *TaARF* members have extra transcripts that undergo 149 AS events, including IR (76), A5SS (29), A3SS (23), ES (20), and MXE (1) events. Thirty-seven (54%) TaARF protein sequences have a DBD, MR, and CTD. Twenty TaARF members are expressed among different organs and tissues. In response to auxin treatment, the expression of nine, 10, and seven *TaARF* members significantly differed in the roots, stems, and leaves of wheat seedlings, respectively. Overall, *TaARF9*, *13*, and *15* responded significantly to auxin treatment in all the three organs. In addition, TaTIR1-TaARF15, 18, 19, and 20-TaIAA12, 13, and 19 were predicted to be interactive proteins, and *TaTIR1*–*TaARF15/19/20*–*TaIAA13* exhibited similar expression patterns at the genetic level. *TaARF15-A.1* is likely involved in the regulation of roots and leaves of *A. thaliana*.

## Author Contributions

LQ, ZC, and JZ conceived and designed the experiments. LQ, WZ, XZ, XL, HG, and YR performed the experiments. LQ analyzed the data. XyL and LQ contributed reagents, materials, and analysis tools. LQ and LZ wrote the manuscript.

## Conflict of Interest Statement

The authors declare that the research was conducted in the absence of any commercial or financial relationships that could be construed as a potential conflict of interest.

## Footnotes

1 http://www.wheatgenome.org/

2 ftp://ftp.ensemblgenomes.org/pub/current/plants/fasta/triticum_aestivum/

3 https://www.ncbi.nlm.nih.gov/

4 https://npsa-prabi.ibcp.fr/cgi-bin/npsa_automat.pl?page=/NPSA/npsa_seccons.html

5 http://string-db.org/

6 http://www.wheatgenome.org/tools-and-resources/sequences

7 http://gsds.cbi.pku.edu.cn/

8 http://www.wheat-expression.com/

9 http://www.tm4.org
