# Supplementary material for: Characterization and Expression Patterns of Auxin Response Factors in Wheat
Source: Front Plant Sci. 2018 Sep 19;9:1395. doi: 10.3389/fpls.2018.01395 (PMC6157421; doi:10.3389/fpls.2018.01395)
Supplement: Supplementary file 1 [file Data_Sheet_1.pdf]

Supplementary Material

Characterization and Expression Patterns of Auxin Response Factors in Wheat

Linyi Qiao, Jun Zheng*, Zhijian Chang*

*** Correspondence:**

Zhijian Chang: czjsxaas@126.com;

Jun Zheng: zhengjsxaas@126.com

Supplementary Figures

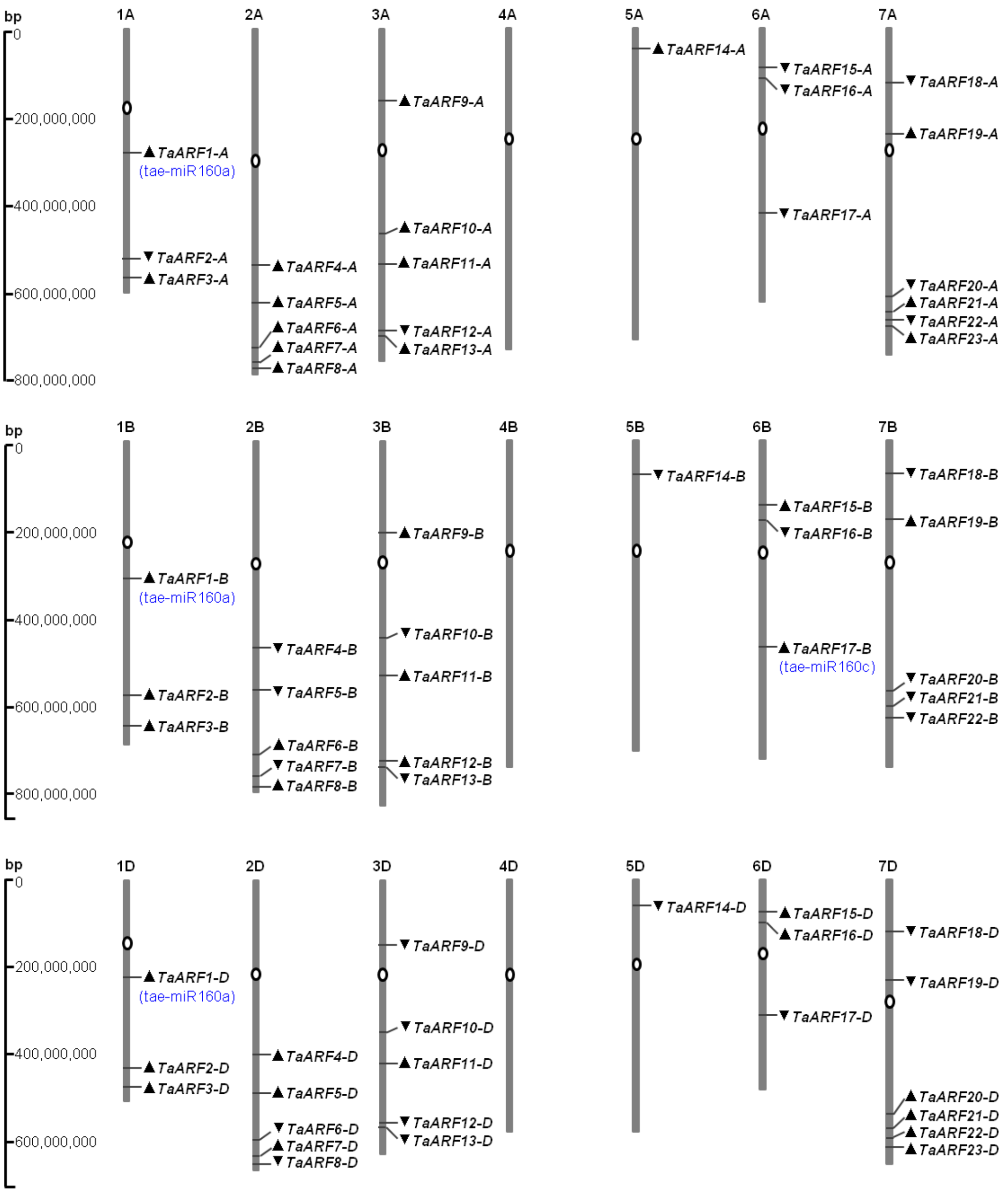

Supplementary Figure 1 | Chromosome distribution of the *TaARF* family. White ovals on the chromosomes (vertical bars) indicate the positions of centromeres. The arrows next to gene names show the direction of transcription. The position of each gene can be estimated using the scale on the left. The chromosome numbers are indicated above each bar. Two *TaARF* members (*TaARF1* and *17*), as the target sequences of microRNAs, are annotated in blue.

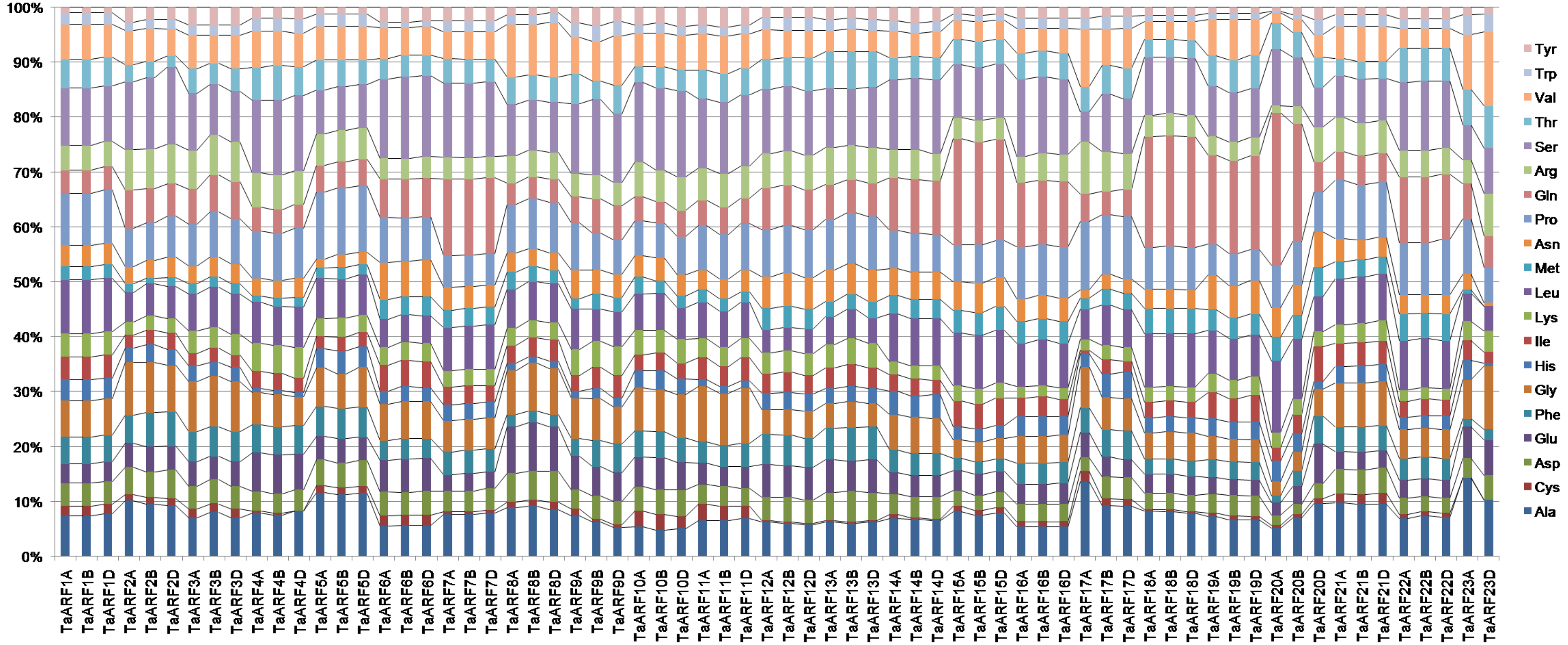

Supplementary Figure 2 | Amino-acid content of MR domains in putative TaARFs. TaARF is the X-axis variable and the corresponding amino acid content is the Y-axis variable. Colored bars represent different amino acidsdomain.

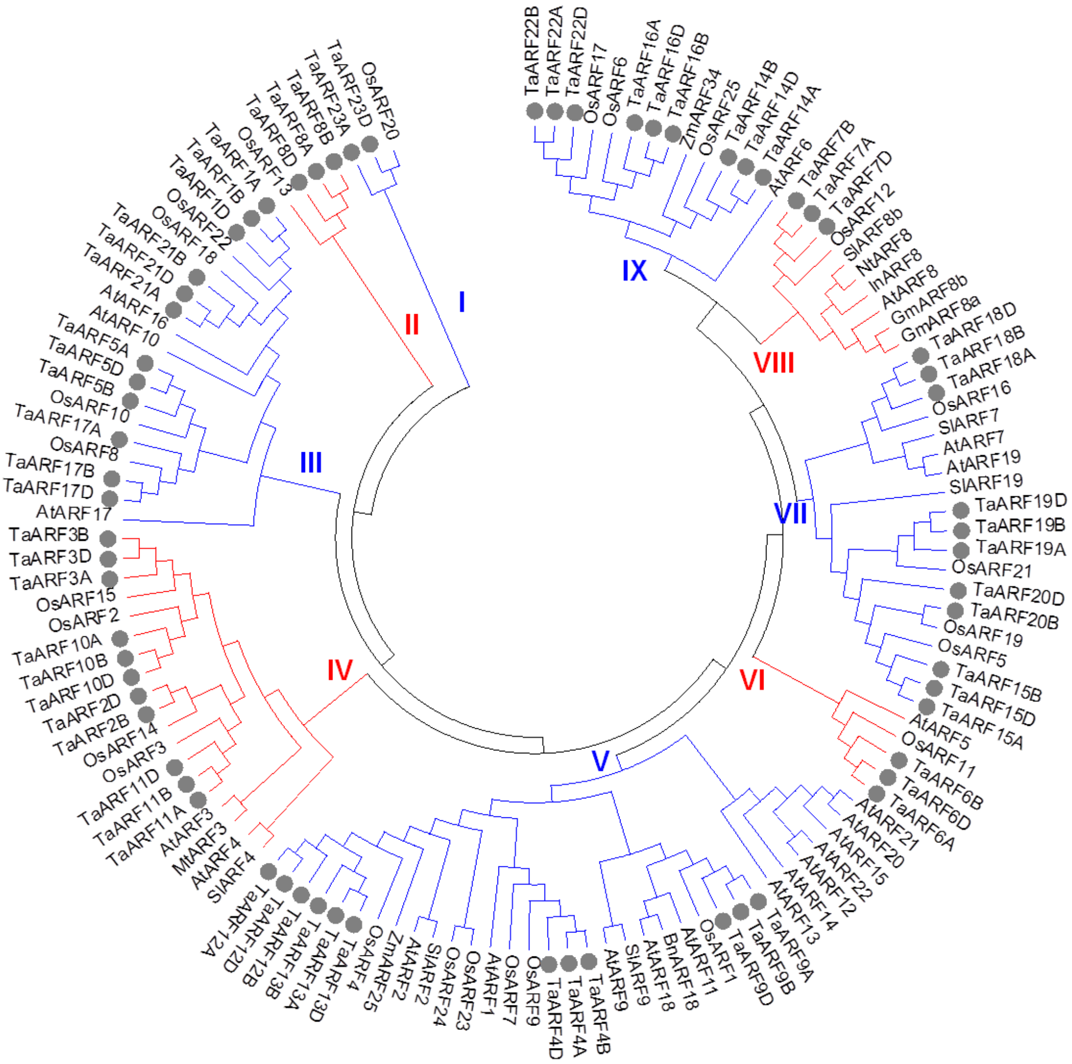

Supplementary Figure 3 | Phylogenetic relationships of ARF proteins between wheat and other species. The full-length AA sequences of 68 ARF homeoalleles in wheat, 23 ARFs in Arabidopsis, 25 ARFs in rice and 14 ARFs in other species were aligned by Clustal X, after which a phylogenetic tree was constructed by the neighbor-joining method with 1,000 bootstrap replicates using MEGA 6.0. Each TaARF protein is labeled by a gray circle. Nine major groups, Groups I to IX, are shown in red and blue. In addition to wheat, the protein IDs of ARFs in other species are listed in Supplementary Table 1.

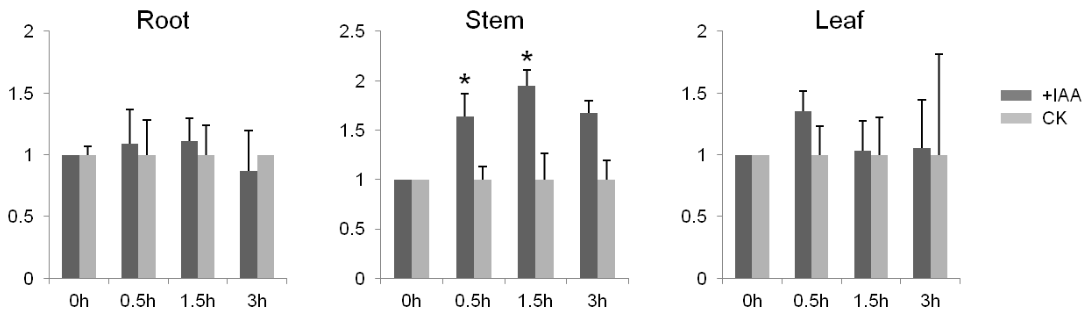

Supplementary Figure 4 | Induction of *TaAux/IAA1* in response to exogenous auxin stimuli in the root, stem and leaf of wheat seedlings. The root, stem and leaf of wheat cultivar "Chinese Spring" at the three-leaf stage were harvested at 0, 0.5, 1.5 and 3 HAT; the plants were treated with 10 μM α-NAA solution or distilled water (mock treatment). The relative expression level of *TaAux/IAA1* (AJ575098; Singla et al. 2006) was measured three times and then normalized to that of the GADPH gene, after which the levels were analyzed using the fold-change method. Paired t-tests were used to detect significant differences in relative expression levels of gene between the auxin treatment and the mock treatment at each time point. The asterisks indicate significant differences, and the error bars indicate the SD.

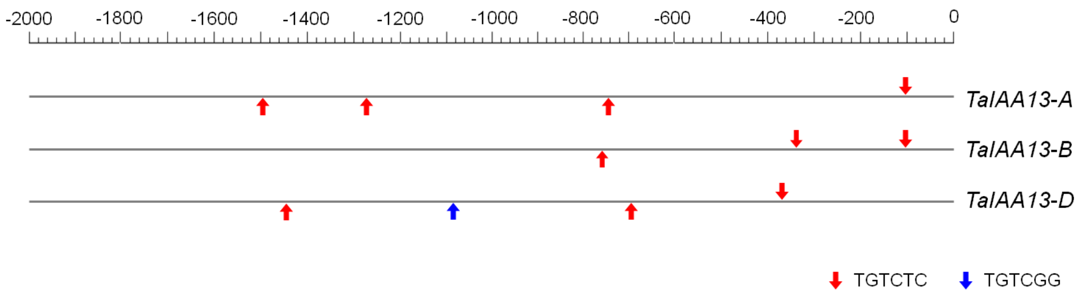

Supplementary Figure 5 | Analyses on auxin response elements (AuxREs) of *TaIAA13*. Putative promoter regions (2000 bp upstream of the start codon) of *TaIAA13* were analyzed for the presence of sequences similar to AuxREs, TGTCTC and TGTCGG, which are displayed in red and blue, respectively.

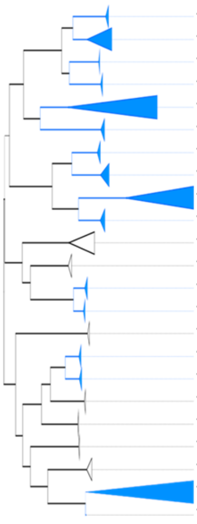

Gene	Chr. location	Predicted duplication pattern	Tt-A	Tt-B	Tu	Aet	Hv	Bd	Os	At
TaARF5	Chr.2L	SD	1	1	1	0	1	1	1	0
TaARF17	Chr.6L		1	1	0	1	0	1	1	0
TaARF1	Chr.1L	SD	1	1	0	1	1	1	1	2
TaARF21	Chr.7L		1	1	1	1	1	1	1	
TaARF23	Chr.7L	SD	1	1	1	1	1	1	1	0
TaARF8	Chr.2L		1	1	0	1	0	1	1	0
TaARF3	Chr.1L	SD	1	1	1	1	1	1	1	0
TaARF10	Chr.3L		1	1	1	1	1	1	1	0
TaARF2	Chr.1L	SD	1	1	1	1	1	1	1	0
TaARF11	Chr.3L		1	1	1	1	1	1	1	0
TaARF9	Chr.3S		1	1	1	1	1	1	1	2
TaARF4	Chr.2L		1	1	1	1	1	1	1	1
TaARF13	Chr.3L	TD	1	1	1	1	1	1	1	1
TaARF12	Chr.3L		1	1	1	1	1			
TaARF6	Chr.2L		1	1	1	1	1	1	2	1
TaARF22	Chr.7L	SD	1	1	1	1	1	1	1	0
TaARF16	Chr.6S		1	1	1	1	1	1	1	0
TaARF14	Chr.5S		1	2	1	1	1	1	1	0
TaARF7	Chr.2L		1	1	1	1	1	1	1	1
TaARF18	Chr.7S		1	1	1	1	1	1	1	2
TaARF19	Chr.7S		1	1	1	1	1	1	1	0
TaARF20	Chr.7L	SD	1	1	1	1	1	1	1	0
TaARF15	Chr.6S		1	1	1	1	1	1	1	0
		Unmapped to TaChr.	0	1	1	1	4	6	2	13
		Total	**25**		**21**	**23**	**25**	**28**	**25**	**23**

Supplementary Figure 6 | Phylogenetic tree and number of homologous genes of 23 *TaARF* family members. The branches of eight pairs of paralogous TaARF members are shown in blue, and the duplication pattern of each pair is predicted. Chromosome position and the number of homologs in seven species are listed in the table on the right.

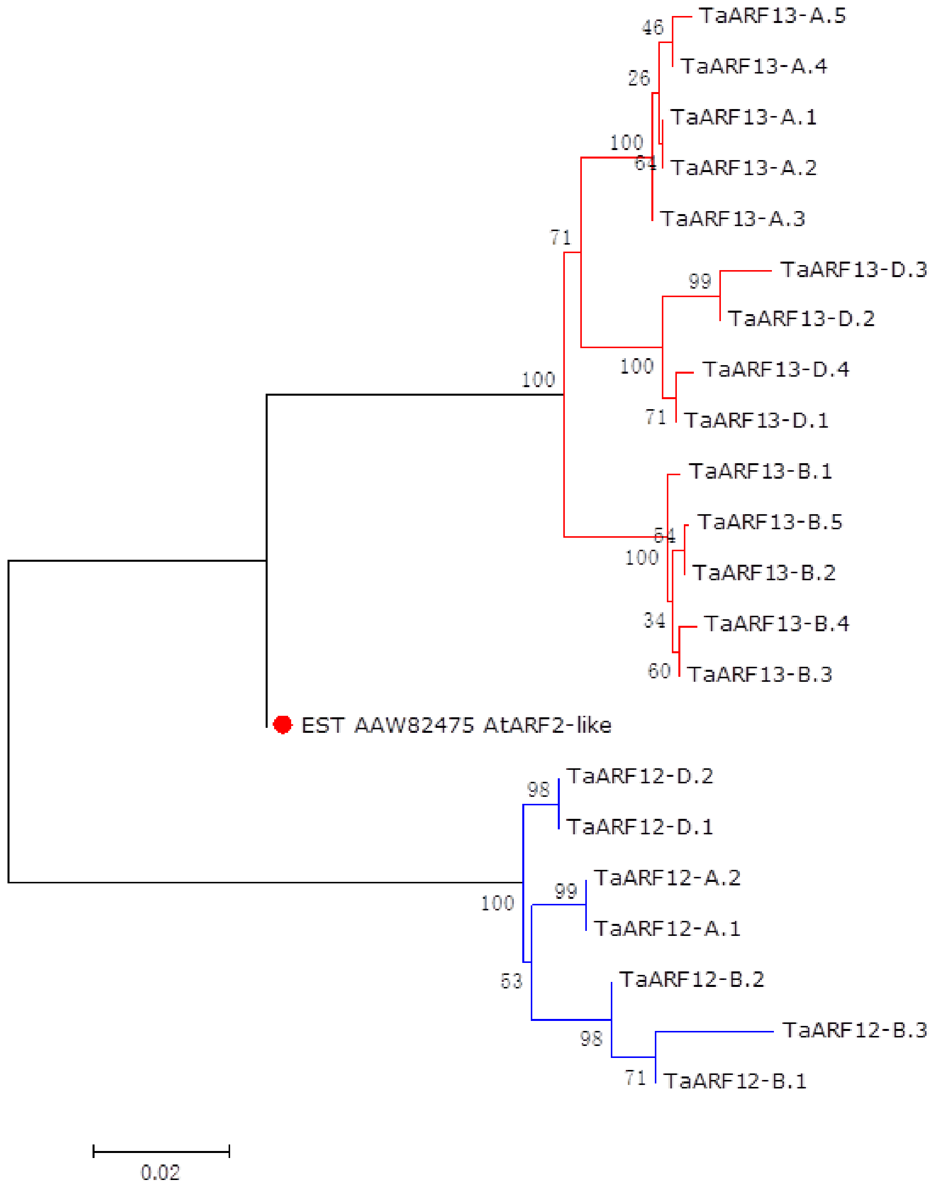

Supplementary Figure 7 | Phylogenetic relationship between expressed sequence tag and TaARF12-13. The expressed sequence tag AAW82475 (AY902381) is labeled by a red circle and the branches of TaARF12 and TaARF13 are showed in blue and red, respectively.

Supplementary Tables

Supplementary Table 1 | ARF protein sequences used in phylogenetic analysis

Species	Gene	Protein ID	Bio-function	Type	Reference
Arabidopsis thaliana	AtARF1	NP_176184	senescence and floral organ abscission	flower development	Ellis et al., 2005
	AtARF2	NP_201006	large-dark green rosette leaves, delayed flowering, thick-long inflorescence, abnormal flower morphology and sterility in early formed flowers, large organ size and delayed senescence and abscission; senescence and floral organ abscission; transgenic plants are more resistant to ABA in the seed germination and primary root growth than the wild type; auxin-mediated leaf longevity; Integration of auxin and BR pathways	root, leaf and flower development	Okushima et al., 2005; Ellis et al., 2005; Vert et al., 2008; Lim et al., 2010; Marin et al., 2010; Wang et al., 2011
	AtARF3/ETT	NP_180942	affects developmental timing and patterning; floral meristem determinacy; organ asymmetry	leaf and flower development	Pekker et al., 2005; Fahlgren et al., 2006; Hunter et al., 2006; Liu et al., 2014
	AtARF4	NP_200853	heteroblasty of adult traits; transformation of abaxial tissues into adaxial ones in all aerial parts	morphogenesis; lateral organs development	Pekker et al., 2005; Hunter et al., 2006
	AtARF5/MP	NP_173414	embryogenic transition; development and differential growth of inflorescence stems; embryonic root formation; embryo-axis formation and auxin-dependent cell expansion; controls DRN transcription in the tips of the embryonic cotyledons; control embryonic vascular tissue formation and root initiation; leaf provascular cells; restoration of vascular patterning during embryogenesis, hypocotyl and root formation, floral bud initiation	embryo, root, leaf and flower development	Hardtke et al., 2004; Weijers et al., 2005; Cole et al., 2009; Kroga et al., 2014; Konishi et al., 2015; Möller et al., 2017; Carey et al., 2017; Wojcikowska et al., 2017
	AtARF6	NP_174323	regulate gynoecium and stamen development; flower maturation; positive regulate phenotypic plasticity of adventitious rooting; tissue reunion in incised inflorescence stems	root, stem and flower development	Nagpal et al., 2005; Wu et al., 2006; Gutierrez et al., 2009; Tabata et al., 2009; Pitaksaringkarn et al., 2013

AtARF7/NPH4	NP_568400	controlling lateral root growth and development; leaf expansion and lateral root formation; cell elongation and auxin-induced gene expression in the seedling hypocotyl; embryo-axis formation and auxin-dependent cell expansion	embryo, root, leaf and flower development	Hardtke et al., 2004; Tatematsu et al., 2004; Wilmoth et al., 2005; Okushima et al., 2005; Weijers et al., 2005; Shin et al., 2007; Okushima et al., 2007; Lee et al., 2009; Goh et al., 2012; Galvan-Ampudia et al., 2014
AtARF8	NP_198518	Negative regulate fruit initiation, pollination and fertilization; regulate gynoecium and stamen development; flower maturation; affect hypocotyl elongation and root growth habit; Developmental Abnormalities; Stimulates Parthenocarpy	embryo, root, flower and fruit development	Tian et al., 2004; Nagpal et al., 2005; Wu et al., 2006; Goetz et al., 2006; Goetz et al., 2007; Jay et al., 2011
AtARF9	NP_194129			
AtARF10	NP_180402	seed germination and post-germination stages	embryo development	Liu et al., 2007
AtARF11	NP_182176			
AtARF12	NP_174691			
AtARF13	NP_174679			
AtARF14	NP_174786			
AtARF15	NP_174784			
AtARF16	NP_567841	regulation of cell wall modification, auxin-regulated root growth inhibition, response to Al stress	root development	Yang et al., 2014
AtARF17	NP_565161	embryo and emerging leaf symmetry anomalies, leaf shape defects, premature inflorescence development, altered phyllotaxy along the stem, reduced petal size, abnormal stamens, sterility, and root growth defects; negative regulate phenotypic plasticity of adventitious rooting	embryo, root, leaf and flower development	Mallory et al., 2005; Gutierrez et al., 2009
AtARF18	NP_567119			
AtARF19	NP_173356	phenotype of leaf and root; leaf expansion and lateral root formation; cell elongation and auxin-induced gene expression in the seedling hypocotyl	embryo, root and leaf development	Weijers et al., 2005; Okushima et al., 2005; Wilmoth et al., 2005; Li et al., 2006
AtARF20	NP_174758			
AtARF21	NP_174701			
AtARF22	NP_174699			

	AtARF23	NP_175062			
rice	OsARF1/(16)	Os01g0236300	crown root formation; gravitropic stimulation enhanced the amount of OsARF1 transcript in the lower, faster-growing flank accompanied by a decrease in the upper flank of gravitropically stimulated rice coleoptiles.	embryo and root development	Waller et al., 2002; Inukai et al., 2005
	OsARF2	Os01g0670800			
	OsARF3	Os01g0753500			
	OsARF4/(2)	Os01g0927600			
	OsARF5	Os02g0141100	-	embryo development	Indoliya et al., 2016
	OsARF6	Os02g0164900	in root	root development	Meng et al., 2009; Shen et al., 2010
	OsARF7	Os02g0557200			
	OsARF8	Os02g0628600	response to exogenous auxin in cultured rice cells; regulator for phosphate homeostasis and root system alteration; regulates root elongation and affects iron accumulation	root development, heavy metal transport; cell development	Yang et al., 2006; Qi et al., 2012; Wang et al., 2014
	OsARF9	Os04g0442000			
	OsARF10	Os04g0519700			
	OsARF11/(5)	Os04g0664400			
	OsARF12//(8)	Os04g0671900			
	OsARF13	Os04g0690600			
	OsARF14	Os05g0515400			
	OsARF15	Os05g0563400			
	OsARF16	Os06g0196700	iron deficiency response	heavy metal transport	Shen et al., 2010; Shen et al., 2015
	OsARF17	Os06g0677800			
	OsARF18/(10)	Os06g0685700			
	OsARF19/(7a)	Os06g0702600	controls rice leaf angles	leaf development	Zhang et al., 2015
	OsARF20	Os07g0183100			

	OsARF21/(7b)	Os08g0520500			
	OsARF22	Os10g0479900			
	OsARF23/(1)	Os11g0523800			
	OsARF24	Os12g0479400	leaf morphology	leaf development	Sakamoto et al., 2013
	OsARF25/(6b)	Os12g0613700			
tomato	SlARF2	NP_001233765	inregulating lateral root formation and flower organ senescence	root, stem, flower and fruit development	Xu et al., 2016; Breitel et al., 2016; Ren et al., 2017
	SlARF4	NP_001233771	abnormal ripening with modified fine pectin structure and tissue architecture in tomato fruit, also regulates sugar metabolism	fruit development	Jones et al., 2002; Guillon et al., 2008; Sagar et al., 2013
	SlARF7	ABO33637	acts as a negative regulator of fruit-set as inhibition of its transcription, to cause parthenocarpic fruit development in transgenic tomato	fruit development	Vriezen et al., 2008; de Jong et al., 2009
	SlARF8	XP_004231633	leaf and flower abscission; Stimulates Parthenocarpy	leaf and flower development	Goetz et al., 2007; Ma et al., 2015
	SlARF9	NP_001234534	regulates cell division activity during early fruit development	fruit development	de Jong et al., 2015
	SlARF19	NP_001234605	leaf and flower abscission	leaf and flower development	Ma et al., 2015
maize	ZmARF25	ADG43159	decreases organ size by affecting cellular proliferation	fruit development	Li et al., 2014
	ZmARF34	AIB05454	regulae auxin-responsive genes in pericycle cells of primary roots	root development	von Behrens et al., 2011
soybean	GmARF8a; GmARF8b	XP_006575466; XP_006596491	nodulation and lateral root development	root development	Wang et al., 2015
Brassica napus	BnARF18	AKN45699	seed weight and silique length in polyploid rapeseed	fruit development	Liu et al., 2015
Ipomoea nil	InARF8	ABN10955	participated in the development of young tissues, especially the shoot apices and flower elements	flower development	Glazinskaa et al., 2014
Medicago truncatula	MtARF3	XP_003593664	regulates compound leaf patterning	leaf development	Peng et al., 2017
Nicotiana tabacum	NtARF8	AFF60411	vegetative growth, floral anthocyanin synthesis, flower colorization, seed production	root and flower development	Zhu et al., 2013

Supplementary Table 2 | Primers used in this study

Primer	Forward sequence (5'-3')	Reverse sequence (5'-3')
rt-TaARF1	GACAGCGTCTCTTGCTTGT	GTGTCTCCCTTGATCCACTT
rt-TaARF2	GTCAACCTCGTAGGCATCTT	CTCTCCGTACACCCAATCTT
rt-TaARF3	GCTCACAACCAAGCAGTTCT	CTGTATGCTCCTCCTTTCCT
rt-TaARF4	GGACGGAAGCTGATTCAGAT	GTATCCGAAGCTGTCAGTGT
rt-TaARF6	CAGCTCATGCTGCATCAAGT	CTTGCTTGACTCCTCTGTCT
rt-TaARF8	GACTGGGATGAATCTGTGGT	GTCTCGGAGATCTTGAGCTT
rt-TaARF9	CTCCATCTTCGTGGTCTACT	CACAGGGACATCTTCTGCTT
rt-TaARF10	GCTTCAGAAAGAAGGTCAACT	CTCTACATCATCATCCCATCT
rt-TaARF11	CACTGTCTGCTGTGGCTAAT	GCTCTTCAGGAACTTCCAGT
rt-TaARF12	CCTTGGAGTCCTTGCAACT	CTCTGCTCTGGTGCCTCTT
rt-TaARF13	GTGAGCTTCGTGTTGGTGTT	AGTTGCAAGGACTCCAAGGT
rt-TaARF14	CGGTCTGCTGAGAAAGTCTT	CCAACCTGTAGTCAGGAGAT
rt-TaARF15	GAGCAGGTTGCAGCTTCTAT	CTGGCTGAAGAGTCATCTGT
rt-TaARF16	CAGAGACAGACGAGGTCTAT	GAGAATCCACCATGGGTACT
rt-TaARF18	GCTGTTTGAGACTGAGGATT	GGTGTGGCAACTGGTTCAAT
rt-TaARF19	CCAAGTACCAGAAGGCAGTT	CCACCTTACAGGATCCAGAT
rt-TaARF20	GTGTGGACATTCCGTCACAT	CCTAGTAGAAGTTGCTGCCT
rt-TaARF21	CCTTGGAGACTTCTTCAGGT	AACGGAACGCAGAGCTTCTT
rt-TaARF22	CTCCAGCACAGGAGTTGTTT	GCGACAAGTCTCTTTGCACT
rt-TaARF23	CAATCATCTCCGTCGAGGTT	GGCTCTTTGACATCCGAGTT
rt-TaIAA12	GAAGGTGGACCTCAAGATGT	CCTGTCCTTGGTTGATGGTT
rt-TaIAA13	CCTCGACTCCTGGATCTCAT	GCTGGCTCCTCTTGTACGA
rt-TaIAA19	GTCGAGGAGAGCGACAAGAT	CTGGAAGCAGCTCTTCCTGT
rt-TaTIR1	GCAGGCTCTTGCAGTAAACT	GTAGAGAACTGACTCCAACAT
rt-15-A1	GGTGCCTTAATGAACAGAAGT	GTAGACTAGCTTCCAACCTAT
rt-15-A2	GGTGCCTTAATGAACAGAAGT	GACTTTCAATAGGATAAGCACAT
rt-GAPDH	CTGCATCATACGATGACATC	TGTCACCGACAAAGTCAGTG
rt-AJ575098	CTATGAGGACACCATTGACT	TTGGGAGCAGGTGGCTTCT
15-A1	ACAAAAAAGCAGGCTTCATGG-CGCAGTCGCCGG	ACAAGAAAGCTGGGTGATTCG-AATTGGTCATAGGGGC
pGATE	GTGGGGACAAGTTTGTACAAA-AAAGCAGGCTTC	GTGGGGACCACTTTGTACAAGA-AAGCTGGGTC
rt-ACTIN2	CAATGGCACTGGAATGGT	ATCTTCAGGCGAAACACG

Supplementary Table 3 | Detailed information on the *TaARF* gene family

Gene	TGACv1-ID	Scaffold	Genome location	FL (bp)	AA	Spliced site	SV-Type
TaARF1A	TRIAE_CS42_1AL_TGACv1_001035_AA0023850.1	1035	1A:278142099-278145724	3822	688	_	
	TRIAE_CS42_1AL_TGACv1_001035_AA0023850.2				688	Intron1 (5'-UTR)	IR
TaARF1B	TRIAE_CS42_1BL_TGACv1_032954_AA0135740.1	32954	1B:310927279-310930970	3888	691	_	
	TRIAE_CS42_1BL_TGACv1_032954_AA0135740.2				691	Intron1 (5'-UTR)	IR
TaARF1D	TRIAE_CS42_1DL_TGACv1_062229_AA0211020.1	62229	1D:217143195-217146858	3860	688	_	
	TRIAE_CS42_1DL_TGACv1_062229_AA0211020.2				657	Intron3	IR*
	TRIAE_CS42_1DL_TGACv1_062229_AA0211020.3				688	Exon1 (5'-UTR), Exon2	ES, A5SS
TaARF2A	TRIAE_CS42_1AL_TGACv1_001359_AA0029280.1	1359	1A:522232504-522234887	2384	402	_	
TaARF2B	TRIAE_CS42_U_TGACv1_641066_AA2084040.1	641066	1B:577607136-577611921	4991	698	_	
TaARF2D	TRIAE_CS42_1DL_TGACv1_063230_AA0225280.1	63230	1D:427149174-427153645	4705	698	_	
TaARF3A	TRIAE_CS42_1AL_TGACv1_001847_AA0035810.1	1847	1A:562316636-562321219	4584	735	_	
	TRIAE_CS42_1AL_TGACv1_001847_AA0035810.2				773	Intron2	IR
TaARF3B	TRIAE_CS42_U_TGACv1_640950_AA2080410.1	640950	1B:649051852-649055145	4880	742	_	
TaARF3D	TRIAE_CS42_1DL_TGACv1_062287_AA0211840.1	62287	1D:469216079-469220112	4380	735	_	
	TRIAE_CS42_1DL_TGACv1_062287_AA0211840.2				780	Intron1	IR
TaARF4A	TRIAE_CS42_2AL_TGACv1_095131_AA0307260.1	95131	2A:532496468-532491859	4610	654	Intron3	IR
	TRIAE_CS42_2AL_TGACv1_095131_AA0307260.2				630	_	
TaARF4B	TRIAE_CS42_2BL_TGACv1_129350_AA0379740.1	129350	2B:466194685-466190155	4531	650	_	
	TRIAE_CS42_2BL_TGACv1_129350_AA0379740.2				654	Intron5	A3SS
	TRIAE_CS42_2BL_TGACv1_129350_AA0379740.3				600	Intron13	IR*
TaARF4D	TRIAE_CS42_2DL_TGACv1_158784_AA0526090.1	158784	2D:394541014-394536499	4516	654	_	
TaARF5A	TRIAE_CS42_2AL_TGACv1_094729_AA0302220.1	94729	2A:623564521-623562375	2370	707	_	
TaARF5B	TRIAE_CS42_2BL_TGACv1_130167_AA0405470.1	130167	2B:563924048-563921743	2358	708	_	
TaARF5D	TRIAE_CS42_2DL_TGACv1_160096_AA0546620.1	160096	2D:480778619-480776280	2398	714	_	
TaARF6A	TRIAE_CS42_2AL_TGACv1_093659_AA0284740.1	93659	2A:724297632-724300670	4990	977	_	
	TRIAE_CS42_2AL_TGACv1_093659_AA0284740.2				977	Intron14 (3'-UTR), Exon15 (3'-UTR)	A5SS, ES
TaARF6B	TRIAE_CS42_2BL_TGACv1_129979_AA0400900.1	129979	2B:714128098-714133008	4911	955	_	

	TRIAE_CS42_2BL_TGACv1_129979_AA0400900.2				955	Intron1 (5'-UTR)	A3SS
	TRIAE_CS42_2BL_TGACv1_129979_AA0400900.3				954	Intron1 (5'-UTR), Intron5	A3SS, A5SS
TaARF6D	TRIAE_CS42_2DL_TGACv1_159381_AA0537490.1	159381	2D:589973755-589978548	4794	955	_	
	TRIAE_CS42_2DL_TGACv1_159381_AA0537490.2				954	Intron5	A5SS
TaARF7A	TRIAE_CS42_2AL_TGACv1_093753_AA0286040.1	93753	2A:755769081-755773752	6105	831	_	
	TRIAE_CS42_2AL_TGACv1_093753_AA0286040.2				831	Intron15 (3'-UTR)	A5SS
	TRIAE_CS42_2AL_TGACv1_093753_AA0286040.3				831	Intron15 (3'-UTR)	IR
	TRIAE_CS42_2AL_TGACv1_093753_AA0286040.4				831	Exon1 (5'-UTR), Intron1 (5'-UTR)	ER, A3SS
TaARF7B	TRIAE_CS42_2BL_TGACv1_129445_AA0383990.1	129445	2B:766562822-766566317	5512	833	_	
	TRIAE_CS42_2BL_TGACv1_129445_AA0383990.2				833	Intron1 (5'-UTR)	IR
TaARF7D	TRIAE_CS42_2DL_TGACv1_157916_AA0501640.1	157916	2D:624362203-624366479	6059	833	_	
TaARF8A	TRIAE_CS42_2AL_TGACv1_094693_AA0301690.1	94693	2A:766152170-766156384	4523	515	_	
TaARF8B	TRIAE_CS42_2BL_TGACv1_129315_AA0378040.1	129315	2B:800260980-800256769	4531	517	_	
TaARF8D	TRIAE_CS42_2DL_TGACv1_158464_AA0519370.1	158464	2D:640998036-641002356	4632	513	_	
TaARF9A	TRIAE_CS42_3AS_TGACv1_211448_AA0690270.1	211448	3A:158468676-158470546	4035	759	_	
TaARF9B	TRIAE_CS42_3B_TGACv1_225719_AA0811440.1	225719	3B:202574731-202577342	2773	445	_	
TaARF9D	TRIAE_CS42_3DS_TGACv1_271898_AA0910450.1	271898	3D:140767006-140768849	3091	484	_	
TaARF10A	TRIAE_CS42_3AL_TGACv1_195685_AA0652720.1	195685	3A:461786295-461789657	5199	666	Intron10 (3'-UTR)	IR
	TRIAE_CS42_3AL_TGACv1_195685_AA0652720.2				666	_	
TaARF10B	TRIAE_CS42_3B_TGACv1_221306_AA0736770.1	221306	3B:440411005-440405651	5355	702	Intron2	IR
	TRIAE_CS42_3B_TGACv1_221306_AA0736770.2				630	_	
TaARF10D	TRIAE_CS42_3DL_TGACv1_251881_AA0885700.1	251881	3D:341512281-341508910	7243	710	_	
TaARF11A	TRIAE_CS42_3AL_TGACv1_194675_AA0637570.1	194675	3A:522276080-522271420	4661	653	Intron10	IR
	TRIAE_CS42_3AL_TGACv1_194675_AA0637570.2				602	_	
TaARF11B	TRIAE_CS42_3B_TGACv1_221509_AA0742350.1	221509	3B:527660773-527654466	6308	660	Intron1	IR
	TRIAE_CS42_3B_TGACv1_221509_AA0742350.2				619	Intron1, Intron10	IR*
	TRIAE_CS42_3B_TGACv1_221509_AA0742350.3				660	_	
TaARF11D	TRIAE_CS42_3DL_TGACv1_249301_AA0844620.1	249301	3D:401898361-401895372	4185	693	_	
TaARF12A	TRIAE_CS42_3AL_TGACv1_193656_AA0616620.1	193656	3A:684040833-684044851	4019	797	_	
	TRIAE_CS42_3AL_TGACv1_193656_AA0616620.2				797	Intron14, Exon15 (3'-UTR)	A5SS, ES

TaARF12B	TRIAE_CS42_3B_TGACv1_224879_AA0802650.1	224879	3B:724737622-724741641	4020	796	_	
	TRIAE_CS42_3B_TGACv1_224879_AA0802650.2				742	Intron2	IR
	TRIAE_CS42_3B_TGACv1_224879_AA0802650.3				589	Exon1~7, Intron7	ES, A3SS
TaARF12D	TRIAE_CS42_3DL_TGACv1_251860_AA0885530.1	251860	3D:547298217-547302231	4015	796	_	
	TRIAE_CS42_3DL_TGACv1_251860_AA0885530.2				796	Intron14 (3'-UTR)	IR
TaARF13A	TRIAE_CS42_3AL_TGACv1_195187_AA0646090.1	195187	3A:688693341-688689311	4322	820	_	
	TRIAE_CS42_3AL_TGACv1_195187_AA0646090.2				824	Intron5	A3SS
	TRIAE_CS42_3AL_TGACv1_195187_AA0646090.3				762	Intron13	IR*
	TRIAE_CS42_3AL_TGACv1_195187_AA0646090.4				762	Intron13~14	IR*
	TRIAE_CS42_3AL_TGACv1_195187_AA0646090.5				871	Intron14	IR
TaARF13B	TRIAE_CS42_3B_TGACv1_223386_AA0781430.1	223386	3B:732619406-732615048	4426	826	_	
	TRIAE_CS42_3B_TGACv1_223386_AA0781430.2				824	Intron14~15	A3SS, A5SS
	TRIAE_CS42_3B_TGACv1_223386_AA0781430.3				762	Intron13	IR*
	TRIAE_CS42_3B_TGACv1_223386_AA0781430.4				893	Intron14	IR
	TRIAE_CS42_3B_TGACv1_223386_AA0781430.5				726	Intron3, Intron14	IR, A3SS
TaARF13D	TRIAE_CS42_3DL_TGACv1_251098_AA0877360.1	251098	3D:552002919-551998802	4440	824	_	
	TRIAE_CS42_3DL_TGACv1_251098_AA0877360.2				886	Intron14	ES
	TRIAE_CS42_3DL_TGACv1_251098_AA0877360.3				826	Intron1	IR
	TRIAE_CS42_3DL_TGACv1_251098_AA0877360.4				726	Exon2~3	IR
TaARF14A	TRIAE_CS42_5AS_TGACv1_393130_AA1268830.1	393130	5A:35627715-35632660	4946	899	_	
	TRIAE_CS42_5AS_TGACv1_393130_AA1268830.2				891	Intron11	A3SS
	TRIAE_CS42_5AS_TGACv1_393130_AA1268830.3				825	Intron13	IR
TaARF14B	TRIAE_CS42_5BS_TGACv1_423450_AA1377000.1	423450	5B:44777246-44782228	4983	891	_	
	TRIAE_CS42_5BS_TGACv1_423450_AA1377000.2				891	Intron1 (5'-UTR)	IR
	TRIAE_CS42_5BS_TGACv1_423450_AA1377000.3				783	Intron1, Intron13	IR*
TaARF14D	TRIAE_CS42_5DS_TGACv1_457945_AA1490960.1	457945	5D:44908071-44913085	5015	892	Intron1	IR
	TRIAE_CS42_5DS_TGACv1_457945_AA1490960.2				892	Intron1 (5'-UTR)	IR
	TRIAE_CS42_5DS_TGACv1_457945_AA1490960.3				900	Intron1~2	IR
	TRIAE_CS42_5DS_TGACv1_457945_AA1490960.4				892	Intron1 (5'-UTR), Intron2 (5'-UTR)	IR
	TRIAE_CS42_5DS_TGACv1_457945_AA1490960.5				837	Intron1~2, Exon4	IR, ES
	TRIAE_CS42_5DS_TGACv1_457945_AA1490960.6				892	Intron2 (5'-UTR)	A3SS

	TRIAE_CS42_5DS_TGACv1_457945_AA1490960.7				892	_	
TaARF15A	TRIAE_CS42_6AS_TGACv1_486530_AA1562300.1	486530	6A:83251488-83258363	6876	1135	_	
	TRIAE_CS42_6AS_TGACv1_486530_AA1562300.2				1040	Intron12	IR*
	TRIAE_CS42_6AS_TGACv1_486530_AA1562300.3				947	Intron12	A5SS
TaARF15B	TRIAE_CS42_6BS_TGACv1_514811_AA1664620.1	514811	6B:140604670-140597812	6859	1131	_	
	TRIAE_CS42_6BS_TGACv1_514811_AA1664620.2				1070	Exon1, Intron1	ES, A3SS
TaARF15D	TRIAE_CS42_6DS_TGACv1_543229_AA1737230.1	543229	6D:65505332-65498504	6829	1129	_	
TaARF16A	TRIAE_CS42_6AS_TGACv1_486005_AA1555510.1	486005	6A:109983416-109987530	4139	779	_	
	TRIAE_CS42_6AS_TGACv1_486005_AA1555510.2				781	Intron11	A5SS
	TRIAE_CS42_6AS_TGACv1_486005_AA1555510.3				781	Intron13 (3'-UTR)	IR
TaARF16B	TRIAE_CS42_6BS_TGACv1_514824_AA1664750.1	514824	6B:175338221-175343059	4839	927	_	
	TRIAE_CS42_6BS_TGACv1_514824_AA1664750.2				927	Exon15 (3'-UTR)	MXE
	TRIAE_CS42_6BS_TGACv1_514824_AA1664750.3				922	Intron14, Exon15	A5SS, ES
	TRIAE_CS42_6BS_TGACv1_514824_AA1664750.4				927	Intron5, Intron14, Exon15 (3'-UTR)	A3SS, A5SS, ES
	TRIAE_CS42_6BS_TGACv1_514824_AA1664750.5				753	Exon1~2, Intron12, Intron14, Exon15	ES, IR*, A5SS
	TRIAE_CS42_6BS_TGACv1_514824_AA1664750.6				863	Intron13~14, Exon15	IR*, A5SS, ES
TaARF16D	TRIAE_CS42_6DS_TGACv1_542633_AA1725840.1	542633	6D:91517263-91522094	4832	927	Intron14	IR
	TRIAE_CS42_6DS_TGACv1_542633_AA1725840.2				863	Intron13~14	IR*
	TRIAE_CS42_6DS_TGACv1_542633_AA1725840.3				887	_	
	TRIAE_CS42_6DS_TGACv1_542633_AA1725840.4				929	Intron14	IR
TaARF17A	TRIAE_CS42_6AL_TGACv1_471402_AA1508260.1	471402	6A:415907841-415906255	1587	528	Intron1	IR*
	TRIAE_CS42_6AL_TGACv1_471402_AA1508260.2				750	_	
TaARF17B	TRIAE_CS42_6BL_TGACv1_499488_AA1584080.1	499488	6B:466695618-466698080	2463	747	_	
TaARF17D	TRIAE_CS42_6DL_TGACv1_526473_AA1684570.1	526473	6D:301268067-301270520	2454	750	_	
TaARF18A	TRIAE_CS42_7AS_TGACv1_570721_AA1839790.1	570721	7A:116872033-116875542	5837	1065	_	
TaARF18B	TRIAE_CS42_U_TGACv1_640742_AA2072180.1	640742	7B:71361800-71367659	5860	1065	_	
TaARF18D	TRIAE_CS42_7DS_TGACv1_622375_AA2038600.1	622375	7D:112046247-112049737	5846	1065	Intron11	IR
	TRIAE_CS42_7DS_TGACv1_622375_AA2038600.2				1030	Exon1, Intron11	ES, IR
	TRIAE_CS42_7DS_TGACv1_622375_AA2038600.3				1047	Exon1, Intron2, Intron11	ES, A5SS, IR

	TRIAE_CS42_7DS_TGACv1_622375_AA2038600.4				1056	Intron1, Exon2, Intron11	A5SS, ES, IR
	TRIAE_CS42_7DS_TGACv1_622375_AA2038600.5				920	_	
	TRIAE_CS42_7DS_TGACv1_622375_AA2038600.6				959	Intron11~13	IR
TaARF19A	TRIAE_CS42_7AS_TGACv1_569633_AA1820700.1	569633	7A:236616443-236620292	6341	1087	Intron11	IR
	TRIAE_CS42_7AS_TGACv1_569633_AA1820700.2				1059	_	
	TRIAE_CS42_7AS_TGACv1_569633_AA1820700.3				991	Intron2, Intron11	IR
	TRIAE_CS42_7AS_TGACv1_569633_AA1820700.4				1049	Intron11, Intron13	A3SS
	TRIAE_CS42_7AS_TGACv1_569633_AA1820700.5				1031	Exon1, Intron1, Intron11	ES, A3SS, IR
TaARF19B	TRIAE_CS42_7BS_TGACv1_591833_AA1923070.1	591833	7B:174574636-174572149	6289	1094	Intron8~9, Intron13	IR, A5SS
	TRIAE_CS42_7BS_TGACv1_591833_AA1923070.2				1066	Intron8~9, Intron12	IR, A5SS, A3SS
	TRIAE_CS42_7BS_TGACv1_591833_AA1923070.3				1069	Intron8~9	IR, A5SS
	TRIAE_CS42_7BS_TGACv1_591833_AA1923070.4				1051	Intron3, Intron7~9, Intron12	IR, A5SS, A3SS
	TRIAE_CS42_7BS_TGACv1_591833_AA1923070.5				1085	Intron4, Intron8~9, Intron12	IR, A5SS, A3SS
	TRIAE_CS42_7BS_TGACv1_591833_AA1923070.6				1091	Intron3, Intron8~9, Intron12	IR, A5SS, A3SS
	TRIAE_CS42_7BS_TGACv1_591833_AA1923070.7				970	Intron2, Intron8~9	IR, A5SS
	TRIAE_CS42_7BS_TGACv1_591833_AA1923070.8				998	Intron2, Intron8~9, Intron12	IR, A5SS
	TRIAE_CS42_7BS_TGACv1_591833_AA1923070.9				1049	Intron8~9, Intron12, Intron14	IR, A5SS
	TRIAE_CS42_7BS_TGACv1_591833_AA1923070.10				1049	Intron8~9, Intron12, Intron14	IR, A5SS
	TRIAE_CS42_7BS_TGACv1_591833_AA1923070.11				1069	_	
	TRIAE_CS42_7BS_TGACv1_591833_AA1923070.12				1038	Exon1, Intron1, Intron8~9, Intron12	ES, IR, A5SS, A3SS
TaARF19D	TRIAE_CS42_7DS_TGACv1_623271_AA2051500.1	623271	7D:222139824-222142560	5183	1036	Intron1, Intron11	IR
	TRIAE_CS42_7DS_TGACv1_623271_AA2051500.2				1064	_	

	TRIAE_CS42_7DS_TGACv1_623271_AA2051500.3				1047	Intron11, Intron13	IR*
	TRIAE_CS42_7DS_TGACv1_623271_AA2051500.4				1036	Intron1, Intron11	IR, A5SS
	TRIAE_CS42_7DS_TGACv1_623271_AA2051500.5				1092	Intron11	IR
	TRIAE_CS42_7DS_TGACv1_623271_AA2051500.6				1005	Intron11~13	IR*
TaARF20A	TRIAE_CS42_7AL_TGACv1_556578_AA1766080.1	556578	7A:608401139-608398712	3077	742	_	
TaARF20B	TRIAE_CS42_7BL_TGACv1_576831_AA1856500.1	576831	7B:565917156-565912467	7915	1174	_	
TaARF20D	TRIAE_CS42_7DL_TGACv1_603661_AA1986870.1	603661	7D:528142251-528137227	5258	483	_	
	TRIAE_CS42_7AL_TGACv1_558468_AA1793590.1				701	_	
TaARF21A	TRIAE_CS42_7AL_TGACv1_558468_AA1793590.2	558468	7A:641580999-641578371	2984	701	Intron1 (5'-UTR)	A5SS
	TRIAE_CS42_7AL_TGACv1_558468_AA1793590.3				701	Intron1 (5'-UTR)	IR
TaARF21B	TRIAE_CS42_7BL_TGACv1_576749_AA1853680.1	576749	7B:602938703-602941206	2850	694	Exon4	ES
	TRIAE_CS42_7BL_TGACv1_576749_AA1853680.2				687	_	
	TRIAE_CS42_7DL_TGACv1_602779_AA1968430.1				695	Intron1 (5'-UTR)	IR
TaARF21D	TRIAE_CS42_7DL_TGACv1_602779_AA1968430.2	602779	7D:556121275-556123822	2911	695	_	
	TRIAE_CS42_7DL_TGACv1_602779_AA1968430.3				674	Intron3	IR*
	TRIAE_CS42_7DL_TGACv1_602779_AA1968430.4				674	Intron1 (5'-UTR), Intron3	IR*
	TRIAE_CS42_7AL_TGACv1_557022_AA1775320.1				818	Exon1~2, Intron2, Intron14	ES, A3SS, IR
	TRIAE_CS42_7AL_TGACv1_557022_AA1775320.2				897	Intron4, Intron14	A5SS, IR
	TRIAE_CS42_7AL_TGACv1_557022_AA1775320.3				909	Intron14	IR
TaARF22A	TRIAE_CS42_7AL_TGACv1_557022_AA1775320.4	557022	7A:657720337-657716471	3867	797	Intron12, Intron14	IR*
	TRIAE_CS42_7AL_TGACv1_557022_AA1775320.5				805	Intron3, Intron14	IR
	TRIAE_CS42_7AL_TGACv1_557022_AA1775320.6				818	Intron1, Intron14	IR
	TRIAE_CS42_7AL_TGACv1_557022_AA1775320.7				904	_	
	TRIAE_CS42_7BL_TGACv1_579596_AA1908810.1				909	_	
TaARF22B	TRIAE_CS42_7BL_TGACv1_579596_AA1908810.2	579596	7B:625301664-625296633	5032	796	Intron12	IR*
	TRIAE_CS42_7BL_TGACv1_579596_AA1908810.3				816	Intron1	IR
	TRIAE_CS42_7DL_TGACv1_602960_AA1972710.1				838	Intron13	A5SS
TaARF22D	TRIAE_CS42_7DL_TGACv1_602960_AA1972710.2	602960	7D:568954531-568949773	4759	906	Intron8	A3SS
	TRIAE_CS42_7DL_TGACv1_602960_AA1972710.3				908	_	
	TRIAE_CS42_7DL_TGACv1_602960_AA1972710.4				817	Exon1, Intron1	ES, A3SS
TaARF23A	TRIAE_CS42_7AL_TGACv1_557656_AA1784590.1	557656	7A:670580194-670578996	1199	371	_	
TaARF23D	TRIAE_CS42_7DL_TGACv1_603629_AA1986520.1	603629	7D:578179047-578177833	1215	354	_	

Supplementary Table 4 | Wheat microRNAs that may regulate *TaARFs*. The sequences of wheat microRNAs that may regulate TaARFs were obtained by retrieving tae-miR target sequence data (downloaded from the Ensembl database) using TaARF CDS as queries with a similarity > 90%, then psRNATarget server (Dai and Zhao, 2011) was used for the validation of bioinformatics with maximum expectation as zero and maximum energy to un-pair the target site (UPE) as 25.0.

TaARF	Ensembl database				psRNATarget server			
	Chr.	Similarity	E-value	Targets ID	miRNA	E-value	UPE	Alignment
TaARF1A	1AL	93.76	0	tae-miR160-22	tae-miR160	0	18.290	miRNA 21 ACCGUAUGUCCCUCGGUCCGU 1<br>::::::::::::::::::::<br>Target 1329 AGGCAUACAGGGAGCCAGGCA 1349
TaARF1B	1BL	97.66	0	tae-miR160-22	tae-miR160	0	18.276	miRNA 21 ACCGUAUGUCCCUCGGUCCGU 1<br>::::::::::::::::::::<br>Target 1338 AGGCAUACAGGGAGCCAGGCA 1358
TaARF1D	1DL	95.32	0	tae-miR160-22	tae-miR160	0	18.256	miRNA 21 ACCGUAUGUCCCUCGGUCCGU 1<br>::::::::::::::::::::<br>Target 1329 AGGCAUACAGGGAGCCAGGCA 1349
TaARF5A	2AL	93.52	0	tae-miR160-24	-			
TaARF5B	2BL	98.91	0	tae-miR160-24	-			
TaARF5D	2DL	95.05	0	tae-miR160-24	-			
TaARF7A	2AL	99.68	0	tae-miR167-49	-			
TaARF7B	2BL	98.71	0	tae-miR167-49	-			
TaARF7D	2DL	98.54	0	tae-miR167-49	-			
TaARF17B	6BL	99.86	0	tae-miR160-20	tae-miR160	0	21.335	miRNA 21 ACCGUAUGUCCCUCGGUCCGU 1<br>::::::::::::::::::::<br>Target 1500 AGGCAUACAGGGAGCCAGGCA 1520
TaARF17D	6DL	100	0	tae-miR160-21	-			

TaARF21A	7AL	99.6	0	tae-miR160-23	-
TaARF21B	7BL	95.32	0	tae-miR160-23	-
TaARF21D	7DL	95.72	0	tae-miR160-23	-
TaARF22A	7AL	96.41	0	tae-miR2022-1179	-
TaARF22B	7BL	96.05	0	tae-miR2022-1179	-
TaARF22D	7DL	99.82	0	tae-miR2022-1179	-

Supplementary Table 5 | Summary statistics for detecting selection of *TaARF15* using branch and branch-site models of PAML

Model	Branch	-lnL	LRT statistic	Parameter estimates	Positive Sites
M0 (one-ratio)	All	-9879.76	-	ω0=0.18	-
Branch model (two-ratio)	Branch a	-9870.16	19.2*	ω0=0.17, ω1=0.66	-
	Branch b1	-9890.05	20.58*	ω0=0.18, ω1=0.99	-
	Branch b2	-9878.63	2.26	ω0=0.19, ω1=0.11	-
	Branch d	-9879.76	0	Not allowed	-
Branch-site model	Branch a-A_{null}	-9623.16	-	p0=0.51, p1=0.29, (p2+p3=0.20) ω0=0.07, ω1=ω2=1	-
	Branch a-*A*	-9716.23	186.14*	p0=0.62, p1=0.36, (p2+p3=0.03) ω0=0.07, ω2=999	717L, 718E, **719N**, 720A, **725D**, 726A, 727R, **729E**, 730I, **731S**, 732T, 734M, 736S
	Branch b1-A_{null}	-9722.58	-	p0=0.49, p1=0.30, (p2+p3=0.21) ω0=0.07, ω1=ω2=1	-
	Branch b1-*A*	-9722.58	0	p0=0.44, p1=0.26, (p2+p3=0.29) ω0=0.07, ω2=2.89	Not allowed

LRT: likelihood ratio test. The asterisks indicate significant differences ($P < 0.05$). The positive selection sites which P>0.95 are listed, and the bold site indicates P>0.99.
